# Human memory B cells show plasticity and adopt multiple fates upon recall response to SARS-CoV-2

**DOI:** 10.1038/s41590-023-01497-y

**Published:** 2023-04-27

**Authors:** Yves Zurbuchen, Jan Michler, Patrick Taeschler, Sarah Adamo, Carlo Cervia, Miro E. Raeber, Ilhan E. Acar, Jakob Nilsson, Klaus Warnatz, Michael B. Soyka, Andreas E. Moor, Onur Boyman

**Affiliations:** 1 Department of Immunology, University Hospital Zurich, Zurich, Switzerland; 2 Department of Biosystems Science and Engineering, ETH Zurich, Basel, Switzerland; 3 Department of Rheumatology and Clinical Immunology, Faculty of Medicine, University of Freiburg, Freiburg, Germany; 4 Center for Chronic Immunodeficiency, Faculty of Medicine, University of Freiburg, Freiburg, Germany; 5 Department of Otorhinolaryngology, Head and Neck Surgery, University and University Hospital Zurich, Zurich, Switzerland; 6 Faculty of Medicine and Faculty of Science, University of Zurich, Zurich, Switzerland

**Keywords:** Immunological memory, Viral infection, Tonsils, Humoral immunity

## Abstract

The B cell response to different pathogens uses tailored effector mechanisms and results in functionally specialized memory B (B_m_) cell subsets, including CD21^+^ resting, CD21^–^CD27^+^ activated and CD21^–^CD27^–^ B_m_ cells. The interrelatedness between these B_m_ cell subsets remains unknown. Here we showed that single severe acute respiratory syndrome coronavirus 2-specific B_m_ cell clones showed plasticity upon antigen rechallenge in previously exposed individuals. CD21^–^ B_m_ cells were the predominant subsets during acute infection and early after severe acute respiratory syndrome coronavirus 2-specific immunization. At months 6 and 12 post-infection, CD21^+^ resting B_m_ cells were the major B_m_ cell subset in the circulation and were also detected in peripheral lymphoid organs, where they carried tissue residency markers. Tracking of individual B cell clones by B cell receptor sequencing revealed that previously fated B_m_ cell clones could redifferentiate upon antigen rechallenge into other B_m_ cell subsets, including CD21^–^CD27^–^ B_m_ cells, demonstrating that single B_m_ cell clones can adopt functionally different trajectories.

## Abstract (web summary)

Boyman and colleagues perform phenotypic and B cell receptor sequencing analysis of memory B cell subsets in severe acute respiratory syndrome coronavirus 2-infected and subsequently vaccinated individuals up to 1 year post-infection to show that single memory B cell clones can adopt different trajectories.

## Main

Upon encounter with cognate antigens, lymphocytes are endowed with the capacity to form memory cells^1,2^. Memory lymphocytes are usually long-lived and provide faster and more vigorous immune responses upon secondary contact with their specific antigen^2^. Some memory cells circulate between blood, secondary lymphoid organs and bone marrow, while others migrate to peripheral tissues and mucosal sites where they can become tissue resident^3^.

Whereas subdivision of labor in terms of tissue homing and effector functions has been well characterized for memory T cells, functionally different subsets also exist for memory B (B_m_) cells. Antigen-stimulated B cells receiving instructive signals from their interaction with helper CD4^+^ T cells can further differentiate in the germinal centers (GCs) of secondary lymphoid organs or using an extrafollicular pathway. B cells that differentiate in the GC undergo affinity maturation through somatic hypermutation (SHM) of the B cell receptor (BCR) following which B cells can become long-lived plasma cells or B_m_ cells^4–6^. Long-lived plasma cells can continuously secrete high-affinity antibodies that are protective against a homologous pathogen^7^, whereas B_m_ cells encode a broader repertoire which allows protection against variants of the initial pathogen after restimulation^8^. Upon antigen reencounter, B_m_ cells differentiate into antibody-secreting plasma cells or reenter GCs where they undergo additional SHM^9^.

B_m_ cells can be subdivided into phenotypically and functionally distinct subsets^10^. In humans, resting B_m_ cells are typically CD21^hi^, and express the tumor necrosis factor (TNF) receptor superfamily member CD27. Additionally, CD21^–^CD27^+^ activated B_m_ cells^11^ might represent a GC-derived population prone to plasma cell differentiation^12^, and CD21^–^CD27^–^ B_m_ cells have been reported in chronic infection, immunodeficiency and autoimmune diseases and are thought to be of extrafollicular origin^13–18^. However, the differentiation path of CD21^–^CD27^+^ B_m_ cells and CD21^–^CD27^–^ B_m_ cells remains ill-defined. Antigen-specific CD21^–^CD27^+^ and CD21^–^CD27^–^ B_m_ cells have been transiently detected after vaccines^12,19–22^ and during infection with certain pathogens^21,23,24^, including severe acute respiratory syndrome coronavirus 2 (SARS-CoV-2) (refs. ^25–29^). CD21^–^CD27^–^ B_m_ cells depend on the transcription factor T-bet for their development^30^, are CD11c^hi^ and express inhibitory coreceptors, such as Fc receptor-like protein 5 (FcRL5) (refs. ^31,32^).

In this article, we studied the kinetics, distribution and interrelatedness of antigen-specific B_m_ cell subsets during acute infection and months 6 and 12 post-infection with SARS-CoV-2 in individuals with mild and severe coronavirus disease 2019 (COVID-19) that have also received SARS-CoV-2 messenger RNA vaccination post-infection, and healthy volunteers before and after SARS-CoV-2-specific vaccination. We found that SARS-CoV-2-specific CD21^–^CD27^+^ activated B_m_ cells and CD21^–^CD27^–^ B_m_ cells were the predominant subsets in circulation during acute infection and upon vaccination. CD21^+^ resting B_m_ cells became prevalent at 6–12 months post-infection. Single-cell RNA sequencing (scRNA-seq) indicated that single B_m_ cell clones adopted different fates upon antigen reexposure.

## Results

### SARS-CoV-2 infection forms a durable B_m_ cell response

**Extended Data Fig. 1:**
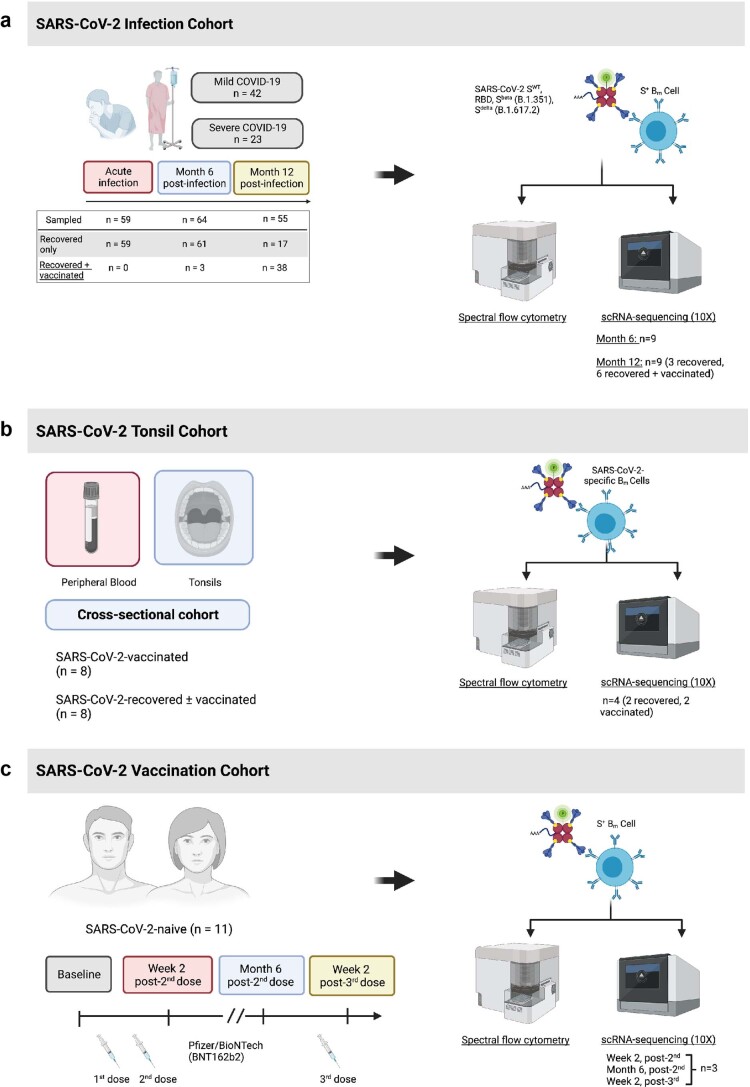
Overview of SARS-CoV-2 cohorts analyzed in this study. **a**, Cohort overview of SARS-CoV-2 Infection Cohort. 65 patients were included and followed-up until month 12 post-infection. 38 patients received SARS-CoV-2 mRNA vaccination during their recovery phase (three between acute infection and month 6, and 35 between month 6 and month 12). All samples were analyzed by flow cytometry and paired month 6 and 12 samples from nine patients also by single-cell RNA sequencing (scRNA-seq). **b**, Cohort overview of SARS-CoV-2 Tonsil Cohort. 16 patients undergoing tonsillectomies for unrelated conditions were included and paired blood and tonsil samples obtained. Eight were vaccinated by SARS-CoV-2 mRNA vaccination only, whereas another eight had recovered from SARS-CoV-2 infection with some of them additionally vaccinated. All samples were analyzed by flow cytometry and paired blood and tonsil samples from four patients also by scRNA-seq. **c**, Cohort overview of SARS-CoV-2 Vaccination Cohort. SARS-CoV-2-naïve healthy controls (*n* = 11) were sampled before their SARS-CoV-2 mRNA vaccination, at week 2 post-second dose, month 6 post-second dose and at week 2 post-third dose. All samples were analyzed by flow cytometry, and paired week 2, month 6 post-second dose and week 2 post-third dose samples from three patients were additionally assessed by scRNA-seq.

We longitudinally studied antigen-specific B_m_ cells in a cohort of 65 patients with COVID-19, 33 females and 32 males, including 42 with mild and 23 with severe disease course, during their acute SARS-CoV-2 infection and at months 6 and 12 post-infection. Of these individuals, 35 received one or two doses of SARS-CoV-2 mRNA vaccination between month 6 and month 12, and three subjects were vaccinated between acute infection and month 6 (Supplementary Table 1 and Extended Data Fig. 1a).

**Fig. 1:**
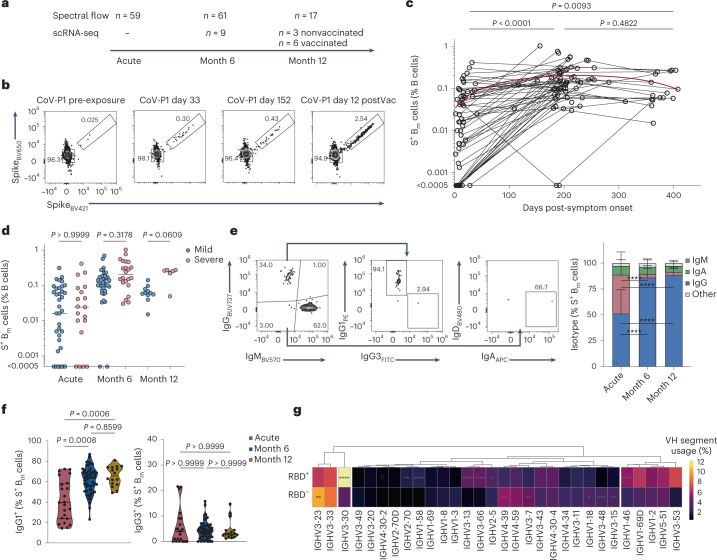
Longitudinal analysis of SARS-CoV-2-specific B_m_ cells post-infection. **a**, SARS-CoV-2-infected patients were analyzed by spectral flow cytometry and scRNA-seq at acute infection and months 6 and 12 post-infection. **b**, Representative flow cytometry plots show percentages of decoy-negative SARS-CoV-2 S^+^ B_m_ cells (gated as in Extended Data Fig. 2a) of patient CoV-P1 pre-exposure to SARS-CoV-2, at days 33 and 152 post-symptom onset and at day 12 post-first dose of SARS-CoV-2 mRNA vaccination (that is, day 166 post-symptom onset). **c**, Frequency of S^+^ B_m_ cells in total B cells was measured by flow cytometry at acute infection (*n* = 59) and months 6 (*n* = 61) and 12 post-infection (*n* = 17). Lines connect samples of same individual. Red line represents fitted second-order polynomial function (*R*^2^ = 0.1932). **d**, Frequency of S^+^ B_m_ cells was measured by flow cytometry and separated by mild (acute, *n* = 40; month 6, *n* = 39; month 12, *n* = 11) and severe COVID-19 (acute, *n* = 19; month 6, *n* = 22; month 12, *n* = 6). **e**, Shown are gating strategy (left) and stacked bar plots (mean + standard deviation; right) of IgG^+^, IgM^+^ and IgA^+^ S^+^ B_m_ cells at indicated timepoints (acute, *n* = 23; month 6, *n* = 52; month 12, *n* = 16). **f**, Violin plots show percentages of IgG1^+^ (left) and IgG3^+^ (right) S^+^ B_m_ cells at indicated timepoints (acute, *n* = 23; month 6, *n* = 52; month 12, *n* = 16). **g**, Heat map represents V heavy (VH) gene usage, in RBD^+^ and RBD^–^ B_m_ cells in scRNA-seq dataset from months 6 and 12. Shown are 30 most frequently used VH segments, sorted by hierarchical clustering, with colors indicating frequencies. Samples in **c**–**f** were compared using Kruskal–Wallis test with Dunn’s multiple comparison, showing adjusted *P* values. Frequencies in **g** were compared using two-proportions *z*-test with Bonferroni’s multiple testing correction. *P* values in **e** and **g** are shown if significant. *P < 0.05, **P < 0.01, ***P < 0.001, ****P < 0.0001. Source data

**Extended Data Fig. 2:**
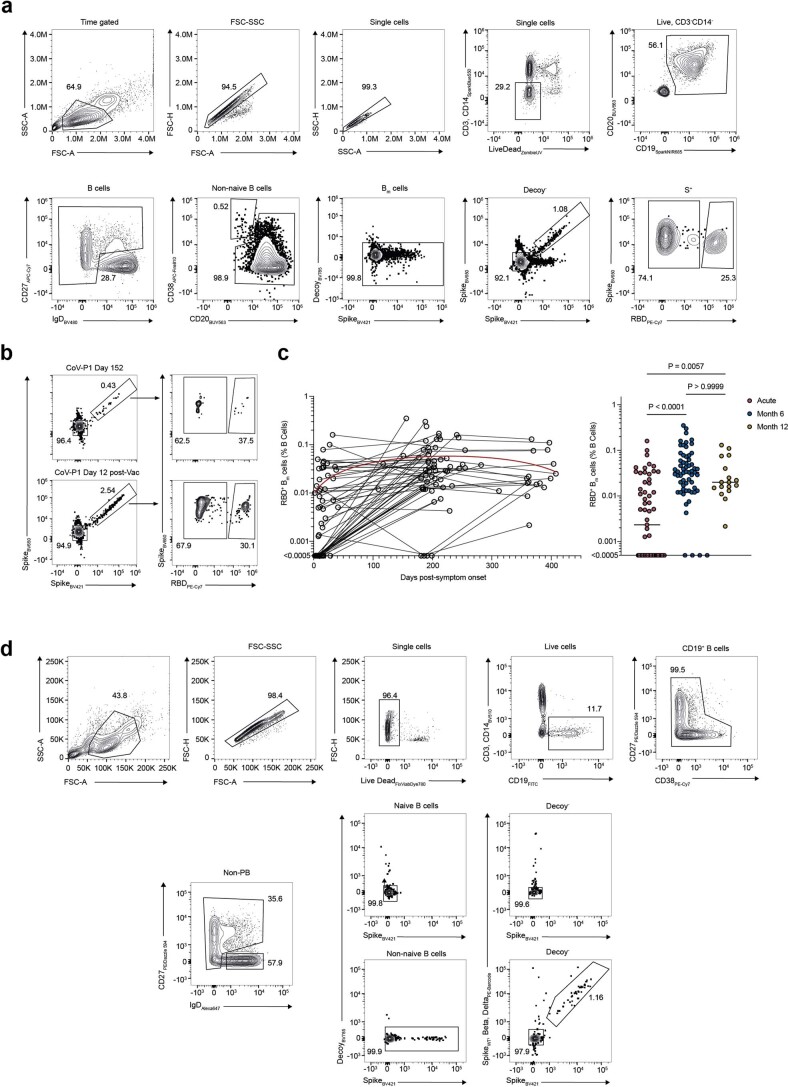
Flow cytometry gating strategies and frequencies of SARS-CoV-2 spike-specific B_m_ cells. **a**, Gating strategy for SARS-CoV-2 spike (S)^+^ and receptor-binding domain (RBD)^+^ B_m_ cells. **b**, Representative flow cytometry plots show gating strategy for RBD^+^ B_m_ cells in patient CoV-P1, as in Fig. 1b. Numbers indicate percentages of parent population. **c**, Frequencies of RBD^+^ B_m_ cells are provided at indicated days post-symptom onset (left), with lines connecting samples of same individual. Red line represents fitted second-order polynomial function (*R*^2^ = 0.1298). Dot plots and medians (right) of frequencies of RBD^+^ B_m_ cells at acute infection (*n* = 59) and month 6 (*n* = 61) and 12 post-infection (*n* = 17). **d**, Sorting strategy for S^+^ and S^–^ B_m_ cells, gated on CD19^+^ non-plasmablasts (non-PB, PB identified as CD38^++^CD27^+^) that were IgD^–^ and/or CD27^+^ and decoy^–^, and for naïve B cells, gated on CD19^+^ non-PB that were IgD^+^CD27^–^ and S^–^ decoy^–^. In **c**, samples were compared using a Kruskal-Wallis test with Dunn’s multiple comparison, with adjusted *P* values shown. Source data

First, we focused on samples from nonvaccinated individuals at acute infection (*n* = 59, day 14 on average after symptom onset), month 6 (*n* = 61, day 202 after symptom onset) and month 12 (*n* = 17, day 374) (Fig. 1a and Supplementary Table 1). SARS-CoV-2-specific B_m_ cells were identified using probes of biotinylated SARS-CoV-2 spike (S) and receptor-binding domain (RBD) protein multimerized with fluorophore-labeled streptavidin (SAV) and characterized using a 28-color spectral flow cytometry panel (Fig. 1b and Extended Data Fig. 2a). We observed a strong increase in the frequency of S^+^ and RBD^+^ B_m_ cells in SARS-CoV-2-infected individuals at months 6 (median 0.14% and 0.033%, respectively) and 12 post-infection (median 0.068% and 0.02%) compared with acute infection (median 0.016% and 0.0023%) (Fig. 1c and Extended Data Fig. 2b,c). Frequencies of S^+^ B_m_ cells were comparable in patients with mild and severe COVID-19 (Fig. 1d). During acute infection S^+^ B_m_ cells were mainly immunoglobulin (Ig)M^+^ and IgG^+^, whereas IgG^+^ B_m_ cells predominated (85–90%) at months 6 and 12 post-infection (Fig. 1e). IgG1 represented the most common subtype (around 65% of S^+^ B_m_ cells at months 6 and 12 post-infection), and between 5% and 10% of S^+^ B_m_ cells were IgA^+^ (Fig. 1e,f).

**Extended Data Fig. 3:**
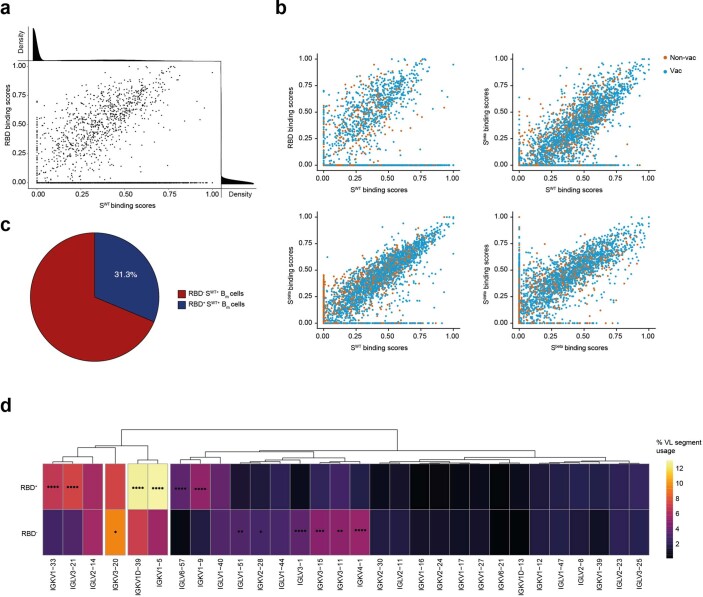
Identification of SARS-CoV-2 S^WT+^, RBD^+^, S^beta+^ and S^delta+^ B_m_ cells using scRNA-seq. **a**, Scatter plot comparing binding scores (LIBRA-Score) was determined from scRNA-seq for S^WT^ and RBD binding, with every dot representing a cell. Density plots indicate count distributions across binding score ranges are shown on top and on the side. **b**, Scatter plots as in **a** display binding scores for S^WT^, RBD, S^beta^ and S^delta^ antigen constructs against each other. **c**, Pie chart show the percentage of S^WT^ binders that also bind RBD in scRNA-seq dataset. **d**, Heatmap displays V light (VL) gene usage in RBD^+^ and RBD^–^ B_m_ cells from scRNA-seq dataset of SARS-CoV-2-infected patients at month 6 and 12 post-infection. VL segments were sorted by a hierarchical clustering. Colors indicate frequency within RBD^+^ and RBD^–^ B_m_ cells. 30 most frequently used segments among RBD^+^ B_m_ cells are shown. In **d**, frequencies were compared using a two-tailed, two-proportions z-test with a Bonferroni-based multiple testing correction. *P* values are shown if significant (p < 0.05). *P < 0.05, **P < 0.01, ***P < 0.001, ****P < 0.0001.

Next, we performed droplet-based scRNA-seq combined with feature barcoding and BCR sequencing (BCR-seq) on sorted S^+^ and S^–^ B_m_ cells isolated from the blood of nine patients with COVID-19 at months 6 and 12 post-infection; three patients were nonvaccinated, and six received SARS-CoV-2 mRNA vaccination between month 6 and month 12 (Extended Data Fig. 2d and Supplementary Table 2). B_m_ cells specific for RBD, wild-type spike (S^WT^) or spike variants B.1.351 (S^beta^) and B.1.617.2 (S^delta^) were identified by SAV multimers carrying specific oligonucleotide barcodes. The majority of S^beta+^, S^delta+^ and RBD^+^ B_m_ cells also recognized S^WT^ (Extended Data Fig. 3a,b). This scRNA-seq approach detected frequencies of about 30% of RBD^+^ B_m_ cells within S^+^ B_m_ cells that were comparable to flow cytometry (Extended Data Figs. 2a and 3c). Analysis of V heavy and light chain frequencies identified several chains enriched in RBD^+^ B_m_ cells compared with RBD^–^ B_m_ cells described to encode RBD-binding antibodies, including *IGHV3-30*, *IGHV3-53*, *IGHV3-66*, *IGKV1-9* and *IGKV1-33* (refs. ^33,34^) (Fig. 1g and Extended Data Fig. 3d). Collectively, these data identify a durable, IgG1-dominated S^+^ B_m_ cell response forming upon SARS-CoV-2 infection.

### Different B_m_ cell subsets form after SARS-CoV-2 infection

**Extended Data Fig. 4:**
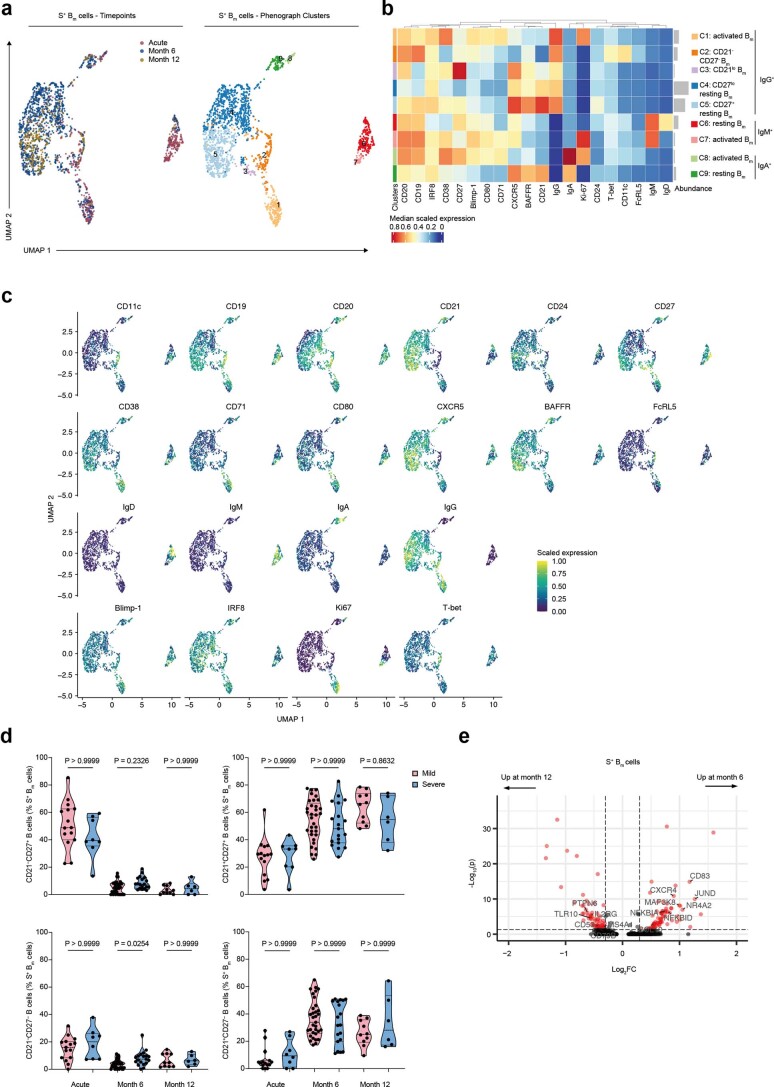
Unsupervised analysis of circulating S^+^ B_m_ cells after SARS-CoV-2 infection. **a**, Uniform manifold approximation and projection (UMAP) plots of S^+^ B_m_ cells are provided during acute SARS-CoV-2 infection and at months 6 and 12, showing samples of nonvaccinated individuals from the SARS-CoV-2 Infection Cohort, subsampled to maximally 25 cells per sample (Acute, *n* = 44; month 6, *n* = 59; month 12, *n* = 17). Cells are colored by timepoint (left) and by clusters identified by PhenoGraph algorithm (right). **b**, Heatmap shows normalized marker expression in the PhenoGraph clusters, with cell numbers for each cluster plotted on the right. The markers were ordered by hierarchical clustering. **c**, UMAP as in **a** was colored by normalized expression of indicated markers. **d**, Violin plots comparing frequencies of CD21^–^CD27^+^, CD21^–^CD27^–^, CD21^+^CD27^+^ and CD21^+^CD27^–^ S^+^ B_m_ cell subsets are separated by timepoints post-infection and mild (acute infection, *n* = 15; month 6, *n* = 33; month 12, *n* = 10) and severe COVID-19 (acute infection, *n* = 8; month 6, *n* = 19; month 12, *n* = 6). **e**, Volcano plot comparing transcript levels in S^+^ B_m_ cells is displayed at month 6 versus 12. X-axis shows log-fold change and y-axis the adjusted *P* values (p < 0.05 was considered significant). In **d**, severities were compared between the same timepoint using a Kruskal-Wallis test with a Dunn’s multiple comparison correction, with adjusted *P* values shown. In **e**, two-sided Wilcoxon rank sum test was used and *P* values corrected by Bonferroni correction. Source data

By using uniform manifold approximation and projection (UMAP) we visualized S^+^ B_m_ cells from the flow cytometry dataset obtained in nonvaccinated post-infection samples and performed a PhenoGraph clustering (Extended Data Fig. 4a–c). UMAP and clustering grouped B_m_ cells by IgG (clusters 1–5), IgM (clusters 6 and 7) and IgA (clusters 8 and 9) expression and revealed a phenotypical shift from acute infection to months 6 and 12 post-infection characterized by increased expression of CD21 on S^+^ B_m_ cells, whereas expression of Blimp-1, Ki-67, CD11c, CD71 and FcRL5 diminished (Extended Data Fig. 4a,c). PhenoGraph clustering identified an IgG^+^CD21^–^CD27^–^ cluster (cluster 2), which was Tbet^hi^CD11c^+^FcRL5^+^, and CD21^–^CD27^+^ clusters characterized by high expression of CD71, Blimp-1 and Ki-67 (clusters 1, 7 and 8) (Extended Data Fig. 4a–c).

**Fig. 2:**
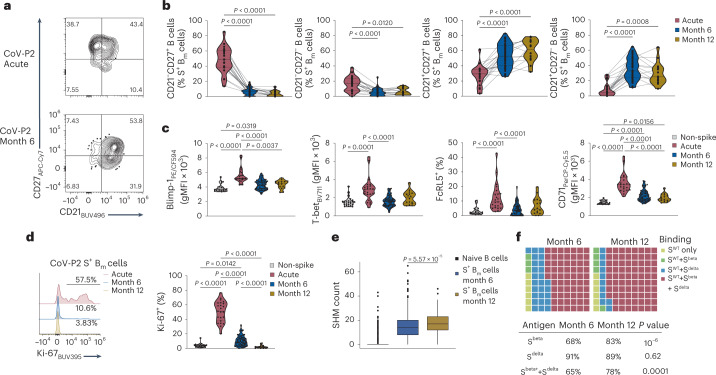
Phenotypic and functional characterization of circulating SARS-CoV-2-specific B_m_ cells post-infection. **a**, CD21 and CD27 expression on S^+^ B_m_ cells during acute infection (top) and month 6 post-infection (bottom) of patient CoV-P2 was determined by flow cytometry. **b**, Violin plots of frequencies of CD21^–^CD27^+^, CD21^–^CD27^–^, CD21^+^CD27^+^ and CD21^+^CD27^–^ cells within S^+^ B_m_ cells are shown at acute infection (*n* = 23) and months 6 (*n* = 52) and 12 post-infection (*n* = 16). Lines connect samples of same individual. Included were only pre-vaccination samples. **c**, Violin plots represent geometric mean fluorescence intensities (gMFI) or percentages of indicated markers in S^+^ B_m_ cells at acute infection (*n* = 23), and months 6 (*n* = 52) and 12 post-infection (*n* = 16), compared with S^–^ B_m_ cells at acute infection (*n* = 23). **d**, Shown are representative histograms of Ki-67 in patient CoV-P2 (left) and violin plots of percentages of Ki-67^+^ S^+^ B_m_ cells compared with S^–^ B_m_ cells (right) at indicated timepoints. **e**, Presented are SHM counts in S^+^ B_m_ cells binding S^WT^, variant S (S^beta^ and S^delta^) or RBD at month 6 (*n* = 634 cells) and month 12 post-infection (*n* = 197 cells; nonvaccinated); SHM counts in naïve B cells (*n* = 1,462) are shown as reference. Box plots show medians, box limits and interquartile ranges (IQRs), with whiskers representing 1.5× IQR and outliers (also applies to subsequent figures). **f**, Waffle plots represent S^WT+^ B_m_ cells binding S^beta^ and S^delta^ in nonvaccinated individuals (*n* = 9 at month 6 and *n* = 3 at month 12 post-infection). Samples in **b**–**d** were compared using Kruskal–Wallis test with Dunn’s multiple comparison correction, showing adjusted *P* values if significant. In **e**, two-sided Wilcoxon test was used with Holm multiple comparison correction. Samples in **f** were compared using two-proportions *z*-test. Source data

The expression changes in CD21 and CD27 on S^+^ B_m_ cells between acute infection and months 6 and 12 post-infection could also be reproduced by manual gating (Fig. 2a). During acute infection S^+^ CD21^–^CD27^+^ B_m_ cells and CD21^–^CD27^–^ B_m_ cells represented on average 48.1% and 16.4% of total S^+^ B_m_ cells, respectively, and they strongly declined at month 6 (6.3% and 5.3%) and month 12 (3.7% and 6.6%) post-infection (Fig. 2b). Conversely, CD21^+^CD27^+^ and CD21^+^CD27^–^ B_m_ cells were prominent at months 6 and 12, amounting to 60.5% and 29.1% of S^+^ B_m_ cells at month 12, respectively (Fig. 2b). These dynamics were comparable in patients with mild and severe COVID-19 (Extended Data Fig. 4d). Expression of Blimp-1, T-bet, FcRL5 and CD71 were increased on S^+^ B_m_ cells during acute infection compared with months 6 and 12 post-infection (Fig. 2c), and S^+^ B_m_ cells underwent strong proliferation during the acute phase (Fig. 2d). S^+^ B_m_ cells continued to show lower but still significantly increased proliferation at month 6, and only returned to background levels at month 12 post-infection (Fig. 2d).

The scRNA-seq dataset identified a significantly increased SHM count in S^+^ B_m_ cells at month 12 compared with month 6 post-infection (Fig. 2e), which correlated with an improved binding breadth, as measured by variant-binding ability of S^WT+^ B_m_ cells (Fig. 2f). At the transcriptional level, S^+^ B_m_ cells at month 6 post-infection upregulated genes associated with B cell activation and recent GC emigration^35^, such as *NKFBIA*, *JUND*, *MAP3K8*, *CXCR4* and *CD83*, compared with S^+^ B_m_ cells at month 12 (Extended Data Fig. 4e). These data showed that SARS-CoV-2 infection induced a stable CD21^+^ B_m_ cell population in the circulation, which continuously matured for more than 6 months.

### Tonsillar S^+^ B_m_ cells undergo tissue adaptation

To extend our analyses to SARS-CoV-2-specific B_m_ cells in the peripheral lymphoid organs, we analyzed paired tonsil and blood samples from a cohort of 16 patients (9 females and 7 males) undergoing tonsillectomy who were exposed to SARS-CoV-2 by infection, vaccination or both. Eight patients were vaccinated against SARS-CoV-2 (analyzed on average at day 144 after last vaccination), whereas the other eight patients were considered SARS-CoV-2-recovered based on a history of SARS-CoV-2 infection or positive anti-nucleocapsid (N) serum antibody measurement, with six of them additionally vaccinated against SARS-CoV-2 (assessed on average at day 118 post-last vaccination) (Extended Data Fig. 1b and Supplementary Table 3).

**Fig. 3:**
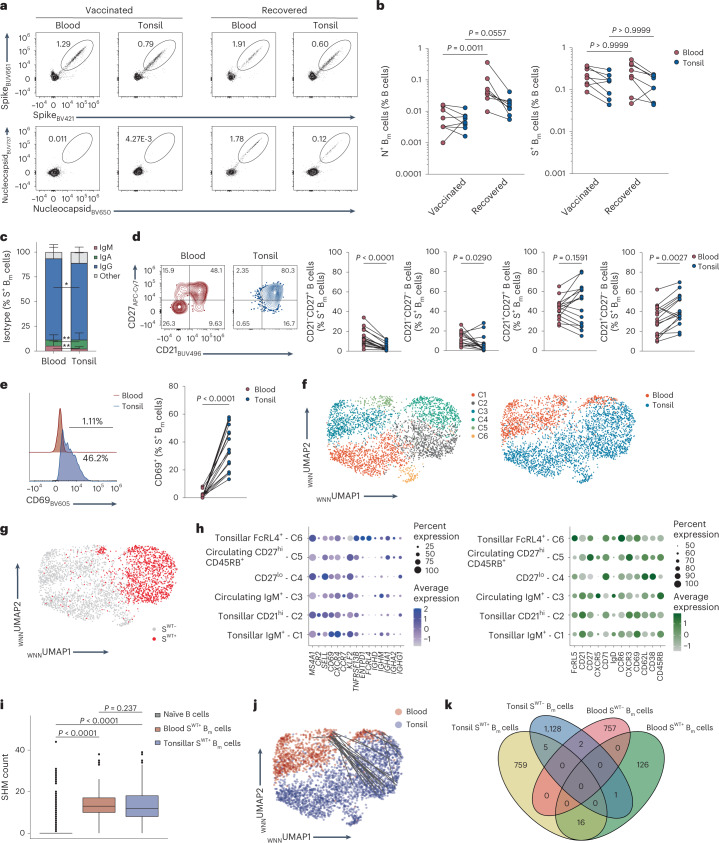
Phenotypic and transcriptional makeup of circulating and tonsillar SARS-CoV-2-specific B_m_ cells post-infection and post-vaccination. **a**, Flow cytometry plots show decoy^–^ S^+^ (top) and nucleocapsid (N)^+^ B_m_ cells (bottom) in paired tonsil and blood samples of a SARS-CoV-2-vaccinated (CoV-T1; left) and SARS-CoV-2-recovered patient (CoV-T2; right). **b**, N^+^ (left) and S^+^ (right) B_m_ cell frequencies were determined in paired blood and tonsils of SARS-CoV-2-vaccinated (*n* = 8) and SARS-CoV-2-recovered individuals (*n* = 8). Lines connect samples of same individual. **c**, Stacked bar plots (mean + standard deviation) represent isotypes in blood and tonsillar S^+^ B_m_ cells from both SARS-CoV-2-vaccinated and SARS-CoV-2-recovered individuals (*n* = 16; also applies to **d** and **e**). **d**, Contour plots show CD21 and CD27 expression on blood and tonsillar S^+^ B_m_ cells of patient CoV-T2 (left) and frequencies of indicated B_m_ cell subsets (right). Lines connect samples of same individual. **e**, Representative CD69 histograms in S^+^ B_m_ cells of patient CoV-T2 (left) and percentages of CD69^+^ S^+^ B_m_ cells (right) in blood and tonsils. **f**,**g**, WNN UMAP of B_m_ cells was derived from scRNA-seq analysis of blood and tonsillar B cells (*n* = 4). B_m_ cells are colored by cluster (**f**, left), tissue origin (**f**, right) or S^WT^ binding (**g**). **h**, Expression of selected genes (left) and surface protein markers (right) are shown in B_m_ cell clusters. **i**, SHM counts are provided for naïve B cells (*n* = 1,607), blood (*n* = 170) and tonsillar S^WT+^ B_m_ cells (*n* = 1,128). **j**, _WNN_UMAP was derived as in **f** and colored by tissue origin. Lines connect shared clones. **k**, Venn diagram shows clonal overlap of S^WT+^ and S^WT–^ B_m_ cells in tonsils and blood from scRNA-seq dataset. Samples in **b** were compared using a Kruskal–Wallis test with Dunn’s multiple comparison correction, in **c**–**e** with a two-tailed Wilcoxon matched-pairs signed-rank test and in **i** with a two-sided Wilcoxon test with Holm multiple comparison correction. *P < 0.05, **P < 0.01. Source data

**Extended Data Fig. 5:**
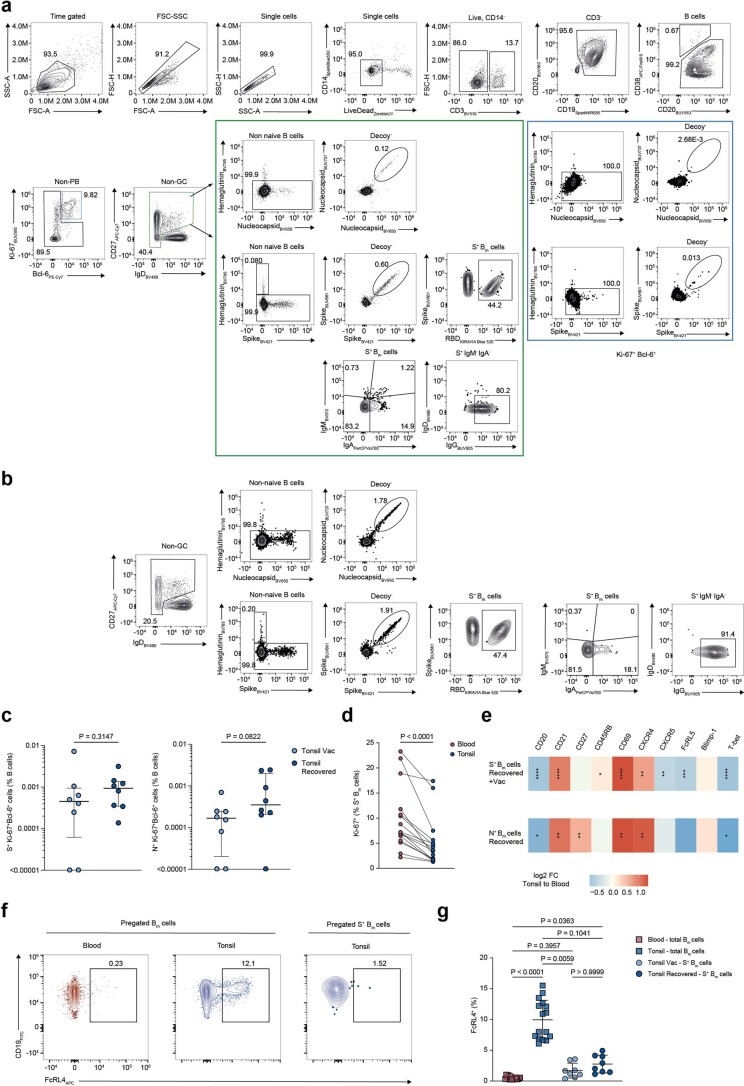
Flow cytometry analysis of tonsillar and circulating SARS-CoV-2-specific B_m_ cells. **a**, Gating strategy is provided for identification of SARS-CoV-2 S^+^ and nucleocapsid (N^+^) germinal center (GC) and B_m_ cells in tonsil from a SARS-CoV-2-recovered and vaccinated individual (CoV-T2). **b**, Gating strategy is shown in a blood sample from the same patient (CoV-T2) as in **a**, with the same gating strategy (including pregating to non-GC cells) applied to tonsil and blood. **c**, Frequency (median ± interquartile range) of S^+^ (left) and N^+^ (right) GC B cells within total B cells are given in tonsils of SARS-CoV-2-vaccinated and in recovered individuals. **d**, Percentages of Ki-67^+^ S^+^ B_m_ cells are provided in paired blood and tonsil samples of SARS-CoV-2-vaccinated and recovered individuals (*n* = 16). **e**, Heatmap of log2-fold change of indicated markers is shown in blood and tonsillar S^+^ B_m_ cells of vaccinated and recovered individuals (top; *n* = 16) and N^+^ B_m_ cells of recovered individuals (bottom; *n* = 8), with red indicating higher expression in tonsils and blue in blood. **f**, Contour plots display FcRL4 expression in tonsillar and blood B_m_ cells – gated as non-PB, non-GC (GC B cells identified as CD38^+^Ki-67^+^), IgD^–^ B cells – and in tonsillar S^+^ B_m_ cells. Numbers indicate percentages of parent population. **g**, Percentages (mean ± SD) of FcRL4^+^ B_m_ cells in paired blood (*n* = 15) and tonsil (*n* = 16) and S^+^ B_m_ cells in tonsil samples, separated by SARS-CoV-2-vaccinated (*n* = 8) and recovered patients (*n* = 8). Frequencies were compared in **c** using two-tailed Mann Whitney test, in **d** and **e** with a two-tailed Wilcoxon matched-pairs signed rank test and in **g** with a Kruskal-Wallis test with a Dunn’s multiple comparison correction, showing adjusted *P* values. In **c** and **g**, all *P* values are shown, in the other graphs adjusted *P* values are shown if significant (p < 0.05). *P < 0.05, **P < 0.01, ***P < 0.001, ****P < 0.0001. Source data

Flow cytometry using the multimer probe approach (Extended Data Fig. 5a,b) identified S^+^ B_m_ cells in the blood and tonsils of both vaccinated and recovered individuals, whereas N^+^ B_m_ cells were enriched only in recovered individuals (Fig. 3a,b). In tonsils, the S^+^ B_m_ cells were less IgG^+^ (77.4% versus 82.1%) and IgM^+^ (2.4% versus 5.5%), but more IgA^+^ (9.1% versus 6%) compared with the circulation (Fig. 3c). Analysis of SARS-CoV-2-specific GC Bcl-6^+^Ki-67^+^ B cells detected a trend towards elevated frequencies of S^+^ and N^+^ GC cells in recovered compared with vaccinated subjects (Extended Data Fig. 5c).

Among the S^+^ B_m_ cell subsets, CD21^–^CD27^+^ B_m_ cells and CD21^–^CD27^–^ B_m_ cells were more frequent in blood, whereas CD21^+^CD27^–^ B_m_ cells were more frequent in tonsils (Fig. 3d). Compared with their circulating counterparts, tonsillar S^+^ and N^+^ B_m_ cells expressed, on average, more CD69, less Ki-67, reduced T-bet and several chemokine receptors differently (Fig. 3e and Extended Data Fig. 5d,e). Very few S^+^ tonsillar B_m_ cells expressed FcRL4 in both vaccinated and recovered individuals (Extended Data Fig. 5f,g).

**Extended Data Fig. 6:**
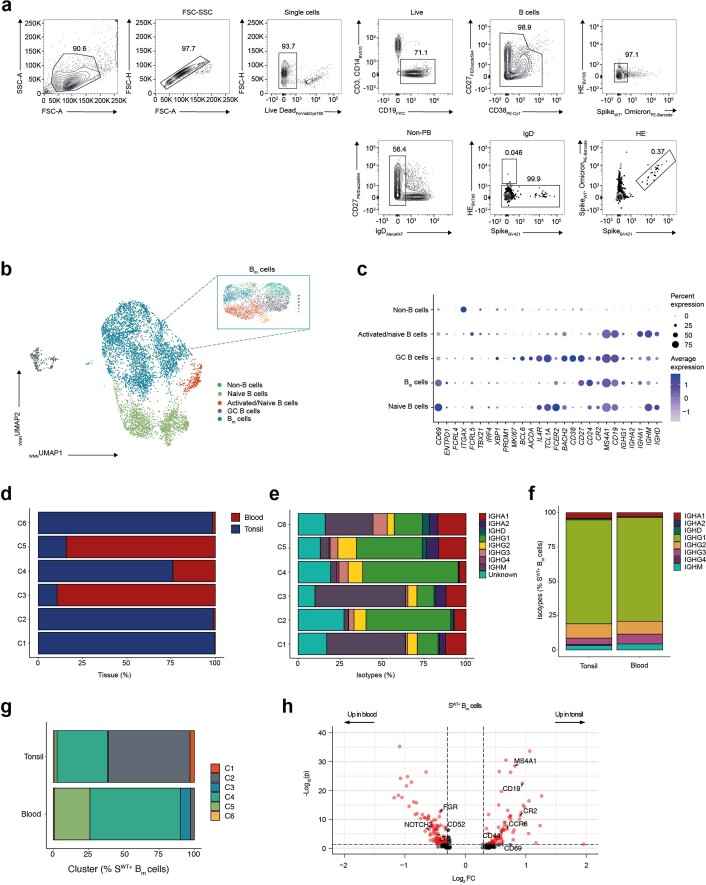
scRNA-seq analysis of B cells in tonsils and blood. **a**, Sorting strategy for SARS-CoV-2 S^+^ B_m_ cells and S^–^ B cells, gated on CD19^+^ non-PB, for scRNA-seq is provided. **b**, Shown is weighted-nearest neighbor (WNN) UMAP analysis from scRNA-seq analysis of fluorescence-activated cell-sorted B cells from paired tonsil and blood samples (SARS-CoV-2-recovered, *n* = 2; SARS-CoV-2-vaccinated, *n* = 2). B cell populations were identified using a WNN clustering and subsequent manual assignment. Identified B_m_ cells (SARS-CoV-2 S^–^ B cells, *n* = 2258; S^WT+^ B_m_ cells, *n* = 1298) were subsequently reclustered as indicated in the box. **c**, Dot plot shows expression of selected genes in main B cell populations. **d–g**, Stacked bar graphs display tissue (**d**) and isotype distribution (**e**) in B_m_ cell clusters, and isotype (**f**) and cluster distribution (**g**) in S^WT+^ B_m_ cells in tonsils and blood. **h**, Volcano plot shows transcript levels in S^WT+^ B_m_ cell in tonsils and blood. X-axis shows log-fold change and y-axis the adjusted *P* values (p < 0.05 was considered significant). In **h**, a two-sided Wilcoxon rank sum test was used, and *P* values corrected by Bonferroni correction.

We performed scRNA-seq combined with feature barcoding, which allowed us to assess surface phenotype and to perform BCR-seq in sorted S^+^ B_m_ cells and S^–^ B cells from paired blood and tonsil samples of four patients (two SARS-CoV-2-recovered and two SARS-CoV-2-vaccinated). Weighted-nearest neighbor (WNN) clustering identified naïve B cells (*IgM*^hi^*IgD*^hi^*FCER2*^hi^), naïve/activated B cells (*IgM*^hi^*IgD*^hi^*FCER2*^hi^*FCRL5*^hi^), GC B cells (*CD27*^hi^*CD38*^hi^*AICDA*^hi^) and B_m_ cells (*IgM*^lo^*IgD*^lo^*CD27*^int^) (Extended Data Fig. 6a–c). Subsequent reclustering of B_m_ cells resolved six clusters (Fig. 3f–h and Extended Data Fig. 6d,e). The S^WT+^ B_m_ cells in the IgG^+^CD27^hi^CD45RB^hi^ cluster (cluster 5) were mainly from blood, in the IgG^+^CD21^hi^ cluster (cluster 2) predominantly tonsillar, while the IgG^+^CD27^lo^ cluster (cluster 4) contained S^WT+^ B_m_ cells from both compartments. The *FCRL4*^hi^*ENTPD1*^hi^*TNFRSF13B*^hi^ cluster (cluster 6) probably represented the FcRL4^+^ B cell subset, and contained very few S^WT+^ B_m_ cells (Fig. 3g,h and Extended Data Fig. 6d–g). Differential gene expression identified higher expression of *CR2*, *CD44*, *CCR6* and *CD69* in tonsillar S^WT+^ B_m_ cells compared with blood S^WT+^ B_m_ cells, whereas the activation-related genes *FGR* and *CD52* were higher in blood S^WT+^ B_m_ cells compared with their tonsillar counterparts (Extended Data Fig. 6h). BCR-seq showed similar SHM counts in S^WT+^ B_m_ cells in blood and tonsils (Fig. 3i). We identified 16 shared S^WT+^ B_m_ cell clones between these compartments (Fig. 3j,k). Taken together, resting antigen-specific B_m_ cells were found in the tonsils after SARS-CoV-2 exposure, and they carried signs of tissue adaptation and clonal connection to their circulating counterparts.

### B_m_ cell subsets reshift following SARS-CoV-2 vaccination

**Fig. 4:**
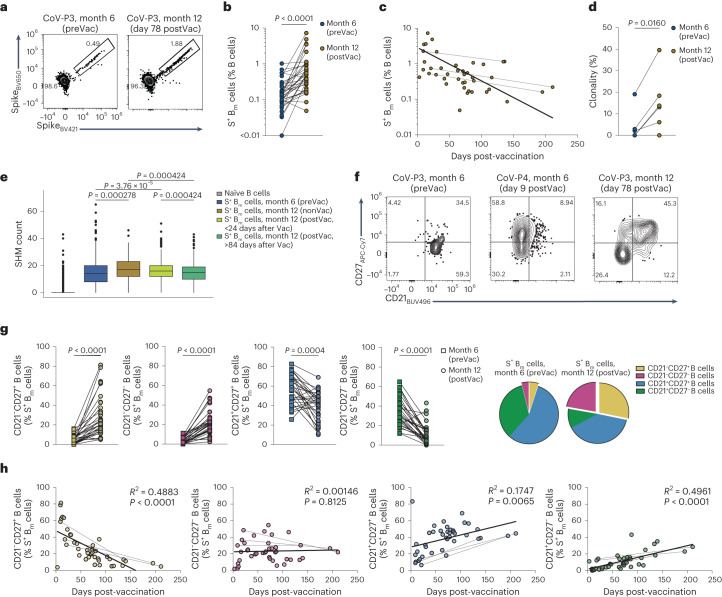
Changes in antigen-specific B_m_ cell subsets following vaccination-induced antigen reexposure. **a**, Representative flow cytometry plots of decoy^–^ S^+^ B_m_ cells are displayed at pre-vaccination (preVac; left; month 6) and day 78 post-vaccination (postVac; right; month 12 post-infection) in patient CoV-P3. **b**, Paired comparison of S^+^ B_m_ cell frequencies within B cells (*n* = 34) was performed at preVac and postVac. **c**, S^+^ B_m_ cell frequencies within B cells (*n* = 41) are plotted against time post-last vaccination. Lines connect paired samples. Semilog line was fitted to data (*R*^2^ = 0.2695). **d**, Clonality of S^+^ B_m_ cells was analyzed preVac and postVac in scRNA-seq dataset. Each dot represents an individual (*n* = 6). **e**, SHM counts of S^+^ B_m_ cells were derived at preVac (*n* = 634 cells), month 12 nonvaccinated (nonVac; *n* = 197 cells), and early (less than 24 days; *n* = 838 cell) and late (more than 84 days; *n* = 1,116 cells) postVac. Naïve B cell (*n* = 1462 cells), served as reference and are the same as in Fig. 2e, as are preVac and nonVac SHM counts. **f**, Representative contour plots of CD21 and CD27 expression on S^+^ B_m_ cells are shown at preVac and day 9 and day 78 postVac. **g**, Frequencies (*n* = 29 pairs; left) and pie charts (right) of indicated S^+^ B_m_ cell subsets are provided at indicated timepoints. **h**, Percentages of S^+^ B_m_ cell subsets are plotted against time post-last vaccination. Lines connect paired samples. Linear regressions are fitted to data. We used a two-tailed Wilcoxon matched-pairs signed-rank test in **b**, **d** and **g**, and two-sided Wilcoxon test in **e**. The Holm–Bonferroni method was used for *P* value adjustment of multiple comparisons. Source data

We probed the B_m_ cell response to antigen reexposure in 35 of the 65 patients with COVID-19 who had received mRNA vaccination between month 6 and month 12 post-infection (Extended Data Fig. 1a and Supplementary Table 1). The frequency of blood S^+^ B_m_ cells was approximately fivefold increased post-vaccination at month 12 compared with pre-vaccination at month 6 post-infection (Fig. 4a,b). Time-resolved analysis identified a peak in the frequency of S^+^ B_m_ cells in the first days post-vaccination, reaching 3% of total B cells on average, followed by a slow decrease in frequency over day 150 post-vaccination (Fig. 4c). The scRNA-seq dataset identified a trend towards increased clonality of S^+^ B_m_ cells in the six patients vaccinated between month 6 and month 12 post-infection when comparing pre-vaccination with post-vaccination (Fig. 4d). Counts of SHM in S^+^ B_m_ cells remained high at month 12 (post-vaccination) compared with month 6 post-infection (pre-vaccination) (Fig. 4e).

Whereas S^+^ B_m_ cells were predominantly resting CD21^+^ B_m_ cells at month 6, vaccination strongly induced the appearance of S^+^ CD21^–^CD27^+^ and CD21^–^CD27^–^ B_m_ cells in blood (Fig. 4f,g). S^+^ CD21^–^CD27^+^ activated B_m_ cells peaked in the first days post-vaccination, followed by a rapid decline over the subsequent 100 days (Fig. 4h). Conversely, the frequency of S^+^ CD21^–^CD27^–^ B_m_ cells rose quickly and remained stable over 150 days post-vaccination, accounting for about 20% of S^+^ B_m_ cells (Fig. 4h).

**Extended Data Fig. 7:**
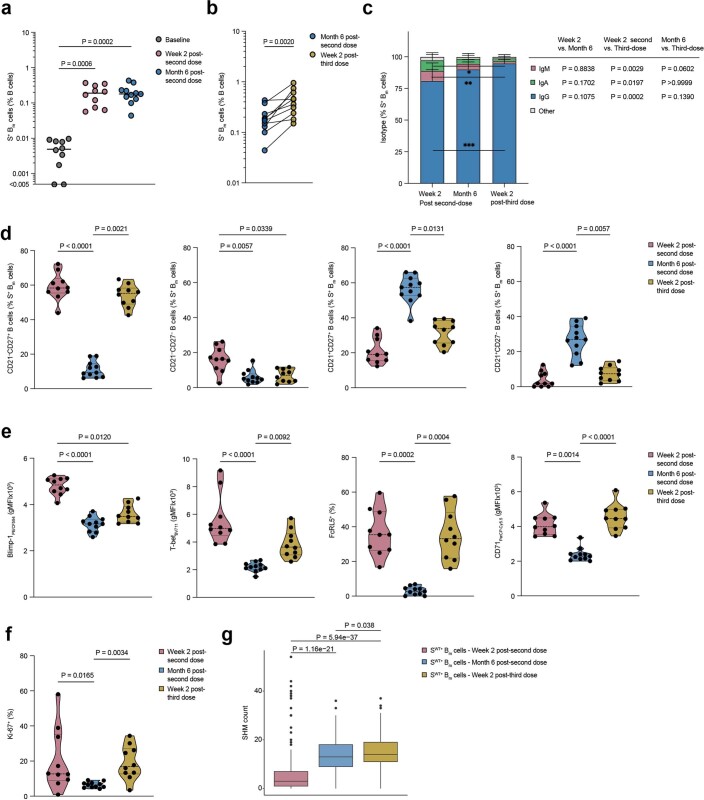
Phenotypic and functional characterization of circulating S^+^ B_m_ cells post-vaccination in SARS-CoV-2-naïve individuals. **a**, Dot plots and medians of frequencies of S^+^ B_m_ cells are provided at baseline (*n* = 10), week 2 post-second dose (*n* = 10) and month 6 post-second dose (*n* = 11). **b**, Paired comparison of S^+^ B_m_ cells frequencies (*n* = 10) is shown at month 6 post-second dose and 11-14 days post-third dose. **c**, Stacked bar plots (mean + SD) show isotypes of S^+^ B_m_ cells at week 2 (*n* = 10) and month 6 (*n* = 11) post-second dose and at week 2 post-third dose (*n* = 10). **d**, Violin plots of frequencies of B_m_ cell subsets of S^+^ B_m_ cells at the indicated time points. **e**, Violin plots of geometric mean fluorescence intensities (gMFI) or percentages of indicated markers in S^+^ B_m_ cells at indicated time points. **f**, Violin plots of percentages of Ki-67^+^ S^+^ B_m_ cells are shown at indicated timepoints. **g**, Comparison of somatic hypermutation (SHM) counts are provided in S^WT+^ B_m_ cells at indicated timepoints (week 2 post-second dose, *n* = 174 cells; month 6 post-second dose, *n* = 271 cells; week 2 post-third dose, *n* = 698 cells). Box plots show median, box limits, and interquartile ranges (IQR), with whiskers representing 1.5x IQR and outliers. Samples in **a** and **c–f** were compared using a Kruskal-Wallis test with Dunn’s multiple comparison correction. Adjusted *P* values are shown if significant (p < 0.05). In **b**, frequencies were compared using a two-tailed Wilcoxon matched-pairs signed rank test. In **g**, two-sided Wilcoxon test was used with Holm multiple comparison correction. Source data

Subsequently, we analyzed S^+^ B_m_ cells in the blood of SARS-CoV-2-naïve individuals (all seronegative for S-specific antibodies) by flow cytometry (*n* = 11, five females and six males) and scRNA-seq (*n* = 3) sampled before their SARS-CoV-2 mRNA vaccination, at days 8–13 (week 2) post-second dose, 6 months after the second dose and days 11–14 post-third dose (Extended Data Fig. 1c and Supplementary Table 4). This revealed a potent induction of S^+^ IgG^+^ B_m_ cells at week 2 post-second dose, which stably persisted to month 6 post-second dose, and the frequency further increased early post-third dose compared with month 6 post-second dose (Extended Data Fig. 7a–c). Antigen-specific B_m_ cells were dominated by CD21^–^CD27^+^ B_m_ cells (around 55% of S^+^ B_m_ cells) and, to a lesser extent, by CD21^–^CD27^–^ B_m_ cells (5–15%) at week 2 post-second dose and post-third dose compared to month 6 post-second dose. Conversely, S^+^ CD21^+^ B_m_ cell subsets became predominant at month 6 post-second dose (Extended Data Fig. 7d).

Flow cytometry analysis of S^+^ B_m_ cells showed an upregulation of Blimp-1 at week 2 post-second dose compared with month 6, and increased expression of T-bet, FcRL5, CD71 and Ki-67 at week 2 post-second dose and post-third dose (Extended Data Fig. 7e,f). The scRNA-seq data showed that SHM counts in S^WT+^ B_m_ cells strongly increased from week 2 post-second (median 3) to month 6 post-second dose (median 13) and even further at week 2 post-third dose (median 14) (Extended Data Fig. 7g). Altogether, these observations indicated that antigen reexposure by SARS-CoV-2 vaccination of SARS-CoV-2-recovered and SARS-CoV-2-vaccinated individuals stimulated S^+^ CD21^–^CD27^+^ and CD21^–^CD27^–^ B_m_ cells.

### S^+^ B_m_ cell subsets show distinct transcriptional profiles

**Fig. 5:**
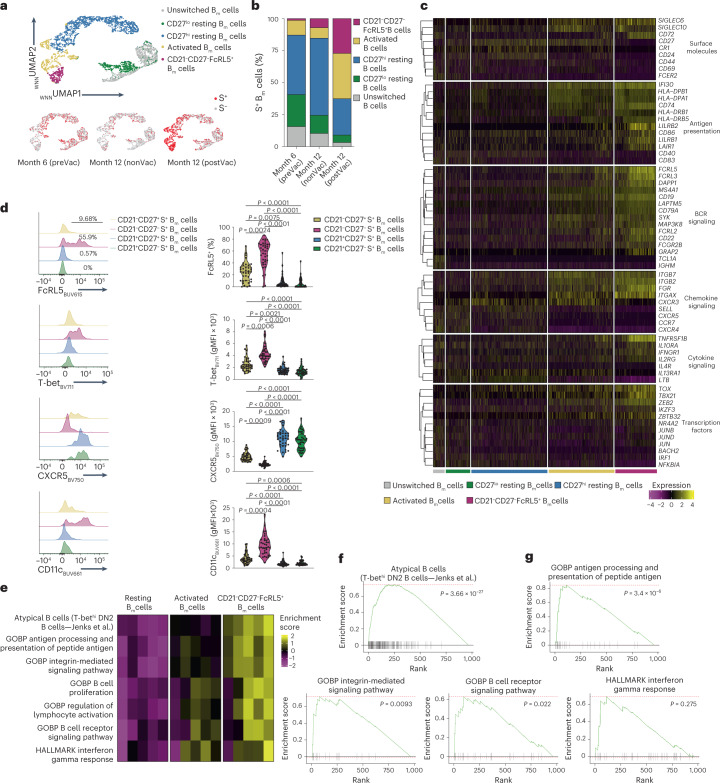
Transcriptional makeup of SARS-CoV-2-specific B_m_ cell subsets. **a**, _WNN_UMAP was derived from scRNA-seq dataset at months 6 and 12 post-infection (*n* = 9) and colored by indicated B_m_ cell subsets (top) and S^+^ and S^–^ separated by month 6 preVac, month 12 nonVac and month 12 postVac (bottom). **b**, Distribution of S^+^ B_m_ cell subsets is provided at month 6 preVac, month 12 nonVac and month 12 postVac. **c**, Heat map shows selected, significantly differentially expressed genes in indicated S^+^ B_m_ cell subsets. Functional groups of genes were ordered by hierarchical clustering. **d**, Representative histograms (left) and violin plots of indicated markers on S^+^ B_m_ cell subsets (right) postVac were derived from the flow cytometry dataset (*n* = 37). **e**, Heat map shows enrichment scores of selected gene sets that are significantly different between CD27^lo/hi^CD21^+^ resting and CD21^–^CD27^–^FcRL5^+^ S^+^ B_m_ cell subsets in a pseudobulk analysis (*n* = 5 individuals). **f**,**g**, GSEA of CD21^–^CD27^–^FcRL5^+^ S^+^ B_m_ cells versus CD21^+^ resting S^+^ B_m_ cells are shown for indicated gene sets. Red dashed lines indicate minimal and maximal cumulative enrichment values. Samples in **d** were compared using Kruskal–Wallis test with Dunn’s multiple comparison correction, showing adjusted *P* values if significant. For **f** and **g**, statistical analysis of the gene set enrichment and variation analyses was performed as outlined in Methods, and all adjusted *P* values are shown. GOPB, Gene Ontology Biological Process. Source data

**Extended Data Fig. 8:**
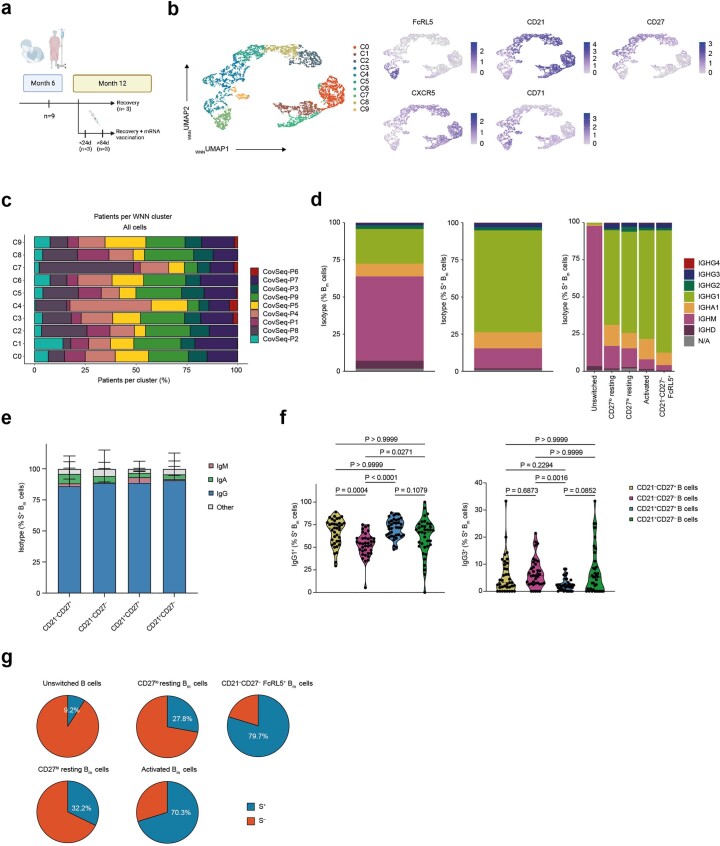
SARS-CoV-2-specific B_m_ cell subset identification by scRNA-seq analysis. **A**, scRNA-seq subcohort of SARS-CoV-2 Infection Cohort. **B**, _WNN_UMAP analysis of B_m_ cells from COVID-19 patients is provided at months 6 and 12 post-infection, colored by clustering based on single-cell transcriptome and cell surface protein levels (left) and by indicated surface protein markers (right). **c**, Stacked bar graphs show single patient contribution to the WNN clusters. **d**, Stacked bar graphs represent isotype and subtype distribution in scRNA-seq dataset on all B cells (left), all S^+^ B_m_ cells (middle) and indicated S^+^ B_m_ cell subsets (right). **e**, Stacked bar graphs (mean + SD) display isotype distribution in S^+^ B_m_ cell subsets in samples of SARS-CoV-2-recovered individuals postVac at months 6 and 12 post-infection from flow cytometry dataset (*n* = 37). **f**, Violin plots of IgG1^+^ (left) and IgG3^+^ percentages (right) are shown in each S^+^ B_m_ cell subset from the same samples as in e. **g**, Pie charts represent percentages of S^+^ B_m_ cells among all cells in scRNA-seq dataset, separated by B_m_ cell subsets. Samples in **f** were compared using a Kruskal-Wallis test with Dunn’s multiple comparison correction, with adjusted *P* values shown. Source data

We used the scRNA-seq of S^+^ and S^–^ B_m_ cells sorted from recovered individuals with and without subsequent vaccination to interrogate the pathways guiding development of different B_m_ cell subsets (Extended Data Fig. 8a). WNN clustering of all sequenced B_m_ cells identified ten clusters that, on the basis of the expression of cell surface markers and Ig isotype, were merged into five subsets annotated as CD21^–^CD27^+^CD71^+^ activated B_m_ cells, CD21^–^CD27^–^FcRL5^+^ B_m_ cells, CD21^+^CD27^–^ resting B_m_ cells, CD21^+^CD27^+^ resting B_m_ cells and unswitched CD21^+^ B_m_ cells (Fig. 5a and Extended Data Fig. 8b,c). Unswitched CD21^+^ B_m_ cells were IgM^+^, whereas the other B_m_ cell subsets expressed mainly IgG, with IgG1 being the dominant subclass (Extended Data Fig. 8d,e). The flow cytometry data further showed that S^+^ CD21^–^CD27^–^ B_m_ cells were enriched in IgG3^+^ compared with CD21^+^CD27^+^ resting B_m_ cells (Extended Data Fig. 8e,f). In the scRNA-seq dataset, CD21^+^CD27^+^ resting B_m_ cells were the main S^+^ B_m_ cell subset at months 6 and 12 post-infection in nonvaccinated individuals, whereas CD21^–^CD27^+^CD71^+^ activated and CD21^–^CD27^–^FcRL5^+^ B_m_ cells became predominant post-vaccination at month 12 post-infection (Fig. 5a,b and Extended Data Fig. 8g).

Analysis of differentially expressed genes indicated that CD21^–^CD27^–^FcRL5^+^ B cells were the most distinctive subset and had high expression of *TBX21* (encoding T-bet), T-bet-driven genes *ZEB2* and *ITGAX* (encoding CD11c), and *TOX* (Fig. 5c). They were also enriched in gene transcripts involved in interferon (IFN)-γ and BCR signaling and showed high expression of integrins *ITGAX*, *ITGB2* and *ITGB7* (Fig. 5c). Moreover, expression of inhibitory receptors, including *FCRL2*, *FCRL3*, *FCRL5*, *SIGLEC6*, *SIGLEC10*, *LAIR1*, *LILRB1* and *LILRB2*, and proteins involved in antigen presentation and processing, such as *HLA-DPA1*, *HLA-DPB1*, *HLA-DRB1*, *HLA-DRB5*, *CD74* and *CD86*, was particularly high in CD21^–^CD27^–^FcRL5^+^ B_m_ cells (Fig. 5c). Several of these differences, such as T-bet, and CD11c, were confirmed at the protein level (Fig. 5d).

Gene set variation and enrichment analysis revealed a strong enrichment of a previously described B cell signature of IgD^–^CD27^–^CXCR5^–^ ‘atypical’ B_m_ cells from patients with systemic lupus erythematosus (SLE)^36^, in our SARS-CoV-2-specific CD21^–^CD27^–^FcRL5^+^ B_m_ cell subset (Fig. 5e,f). Gene sets involved in antigen presentation and integrin-mediated signaling, as well as B cell activation, BCR and IFN-γ signaling were enriched in CD21^–^CD27^–^FcRL5^+^ B_m_ cells compared with other B_m_ cell subsets (Fig. 5e,g). In summary, the data showed that S^+^ CD21^–^CD27^–^FcRL5^+^ B_m_ cells carried a very distinct transcriptional profile, similar to certain B cells found in autoimmunity.

### BCR-seq reveals clonal branching of S^+^ B_m_ cell subsets

**Extended Data Fig. 9:**
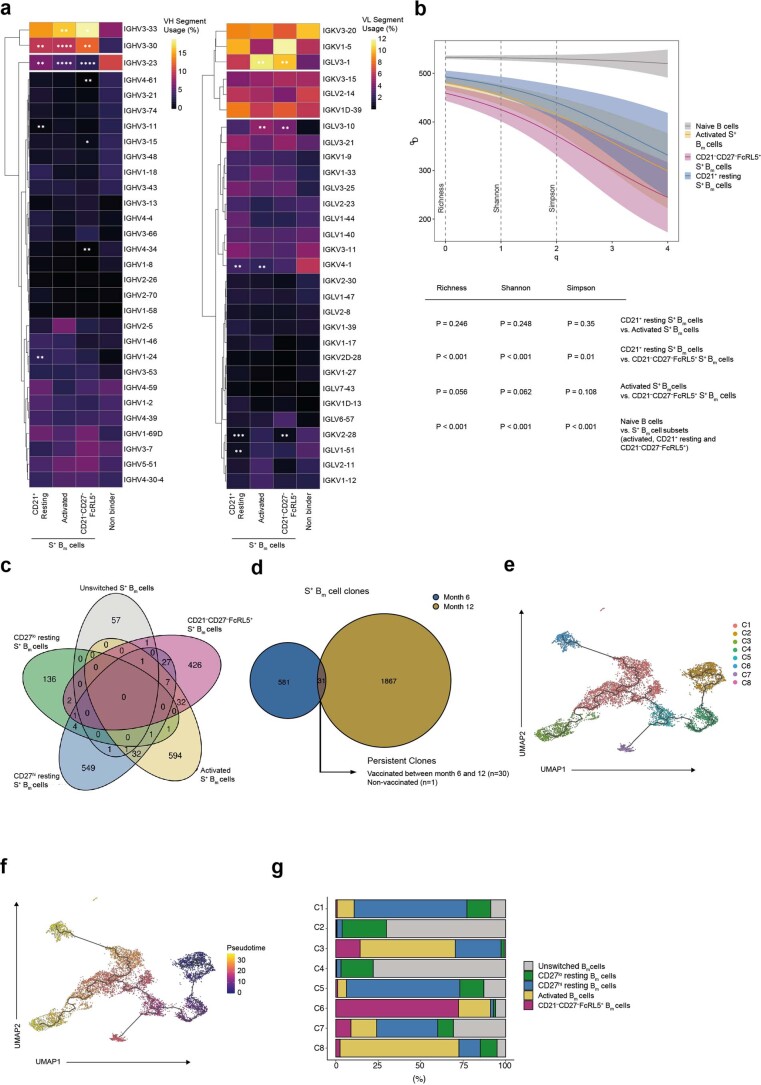
scRNA-seq B cell receptor (BCR) repertoire and Monocle analysis. **a**, Heatmap compares V heavy (VH; left) and VL (right) gene usage in indicated S^+^ B_m_ cell subsets and S^–^ B_m_ cells (non-binders) from scRNA-seq data of SARS-CoV-2-infected patients at months 6 and 12 post-infection. VH/VL were clustered hierarchically, with colors indicating frequencies. 30 most frequently used segments in resting B_m_ cells are displayed. Asterisks indicate significantly different segment usage between S^–^ and the respective S^+^ B_m_ cell subsets. **b**, Hill numbers diversity curves show clonal diversities over a range of diversity orders for indicated S^+^ B_m_ cell subsets and naïve B cells. Mean diversity index (line) and confidence intervals (transparent shadings) are shown. *P* values for different comparisons are given below. **c**, Venn diagram shows clonal overlap of SARS-CoV-2-specific clones in different B_m_ cell subsets. **d**, Venn diagram displays clonal overlap of SARS-CoV-2-specific clones at months 6 and 12 post-infection. **e** and **f**, UMAP represents Monocle 3 analysis on all B_m_ cells in scRNA-seq dataset, colored by clusters identified (**e**) or pseudotime annotation (**f**). The beginning of pseudotime was manually set inside the partition with mostly unswitched B cells. **g**, Stacked bar graphs show contribution of total B_m_ cell subsets to Monocle clusters. In **a**, *P* values were calculated by fitting a linear model to count data using edgeR. Genewise statistics were conducted using empirical Bayes quasi-likelihood F-tests. *P* values are provided if significant (p < 0.05) between the S^–^ and S^+^ B_m_ cell subsets. No VH or VL chain segments were significantly differentially used between S^+^ B_m_ cell subsets. In **b**, significant differences between groups were determined by constructing a bootstrap delta distribution for each pair of unique values between groups. *P < 0.05, **P < 0.01, ***P < 0.001, ****P < 0.0001.

Comparison of V heavy and light chain usage within S^+^ B_m_ cell subsets in the scRNA-seq data from SARS-CoV-2-recovered individuals (months 6 and 12 post-infection) revealed very similar chain usage in S^+^ CD21^+^ resting (CD21^+^CD27^+^ and CD21^+^CD27^–^ combined), CD21^–^CD27^+^CD71^+^ activated and CD21^–^CD27^–^FcRL5^+^ B_m_ cells (Extended Data Fig. 9a). BCR diversity was slightly reduced in S^+^ CD21^–^CD27^–^FcRL5^+^ compared with S^+^ CD21^+^ resting B_m_ cells (Extended Data Fig. 9b). BCR-seq detected shared clones mostly between S^+^ CD21^+^CD27^+^ and CD21^–^CD27^+^CD71^+^ activated B_m_ cells, as well as the CD21^–^CD27^–^FcRL5^+^ B_m_ cell subset (Extended Data Fig. 9c), indicating that S^+^ B_m_ cell subsets had comparable BCR repertoires, although the depth of our analysis was restricted by low cell numbers.

**Fig. 6:**
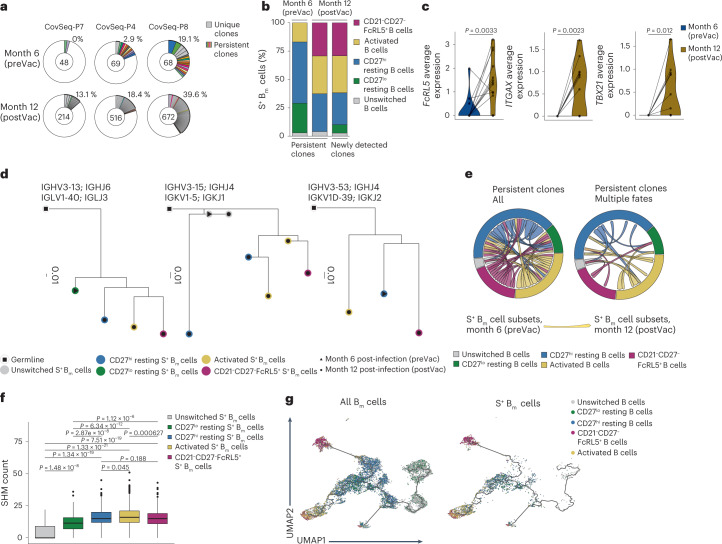
Temporal analysis of individual SARS-CoV-2-specific B_m_ cell subsets. **a**, Donut plots of BCR sequences of S^+^ B_m_ cells in three representative patients preVac and postVac. Numbers inside donut plots represent counts of S^+^ B_m_ cells. Gray slices indicate individual clones found at one timepoint only, whereas persistent clones found at both timepoints are labeled by the same color. White areas represent BCR sequences found in single cells only. Slice sizes correspond to clone sizes. Percentages indicate frequencies of clonally expanded cells. **b**, Distribution of S^+^ B_m_ cell subsets in persistent and newly detected clones is shown at indicated timepoints. **c**, Average expression of indicated genes was derived at preVac and postVac in persistent S^+^ B_m_ cell clones that contained at least one CD21^–^CD27^–^FcRL5^+^ S^+^ B_m_ cell (*n* = 14 clones). **d**, Exemplary dendrograms (IgPhyML B cell trees) display different persistent B_m_ cell clones at months 6 (triangles) and 12 (dots) post-infection. Colors represent B_m_ cell subsets. Germline sequences, inferred by the Immcantation pipeline, are shown in white (squares). Branch lengths represent mutation numbers per site between each node. VH and V light (VL) genes are indicated on top of dendrograms. **e**, Circos plots of all persistent S^+^ B_m_ cell clones (left) and those adopting multiple B_m_ cell fates (right) are shown, with arrows connecting cells of months 6 with 12 and colored according to B_m_ cell phenotype at month 12. **f**, SHM counts were calculated in indicated S^+^ B_m_ cell subsets (unswitched, *n* = 53; CD27^lo^ resting, *n* = 122; CD27^hi^ resting, *n* = 535; activated, *n* = 713; CD21^–^CD27^–^FcRL5^+^, *n* = 531). **g**, UMAPs represent Monocle 3 analysis of all B_m_ cells (left) and S^+^ B_m_ cells (right). Colors indicate B_m_ cell subsets. Black lines indicate trajectory. Samples were compared using paired *t*-test (**c**) or two-sided Wilcoxon test (**f**). Holm–Bonferroni method was used for *P* value adjustment of multiple comparisons. Source data

Longitudinal tracking of S^+^ B_m_ cell clones between month 6 and month 12 post-infection identified 30 persistent clones in individuals vaccinated during that period (Fig. 6a and Extended Data Fig. 9d). At month 6 post-infection (pre-vaccination), 80% of those 30 clones had a CD21^+^ resting B_m_ cell phenotype (Fig. 6b), whereas at month 12 post-infection (post-vaccination) 32% of persistent B_m_ clones showed a CD21^–^CD27^+^CD71^+^ and 28% a CD21^–^CD27^–^FcRL5^+^ B_m_ cell phenotype. The S^+^ B_m_ cell subset distribution of newly detected clones (*n* = 1,357 clones) at month 12 post-infection (post-vaccination) was comparable to the persistent clones (Fig. 6b). Between month 6 and month 12 post-infection, persistent B_m_ cell clones upregulated genes associated with CD21^–^CD27^–^FcRL5^+^ B_m_ cells, including *TBX21*, *ITGAX* and *FCRL5* (Fig. 6c). In addition, reconstruction of clonal lineage trees and visualizing persistent S^+^ B_m_ cell clones in a circos plot indicated that individual B_m_ cell clones acquired different B_m_ cell fates; for example, a given clone was of a CD21^+^CD27^–^ resting phenotype at month 6 and adopted CD21^+^CD27^+^ resting, CD21^–^CD27^+^CD71^+^ or CD21^–^CD27^–^FcRL5^+^ B_m_ cell phenotype at month 12 post-infection (post-vaccination) (Fig. 6d,e).

SHM counts were low in unswitched S^+^ CD21^+^ B_m_ cells, slightly higher in CD21^+^CD27^–^ resting B_m_ cells, and high by comparison in CD21^+^CD27^+^ resting, CD21^–^CD27^+^CD71^+^ activated and CD21^–^CD27^–^ B_m_ cells (Fig. 6f). Pseudotime-based trajectory analysis using Monocle 3 in our scRNA-seq dataset (Extended Data Fig. 9e–g) and visualization of B_m_ cells on the Monocle UMAP space identified two branches, which strongly separated CD21^–^CD27^+^CD71^+^ activated and CD21^–^CD27^–^FcRL5^+^ B_m_ cells, both branching out from CD21^+^ resting B_m_ cells (Fig. 6g and Extended Data Fig. 9e). Collectively, these observations indicated that individual S^+^ B_m_ cell clones could adopt different B_m_ fates post-vaccination in SARS-CoV-2-recovered individuals.

## Discussion

In this study, we demonstrated that individual clones of SARS-CoV-2-specific B_m_ cells harbored the capacity to follow phenotypically and functionally different trajectories after antigen reexposure, becoming CD21^–^CD27^+^, CD21^–^CD27^–^ or CD21^+^CD27^+/–^ B_m_ cells.

The transient occurrence of vaccine-specific CD21^–^CD27^–^ B_m_ cells has been described during responses to the influenza vaccine^12,20^, with one study reporting this B_m_ cell subset in de novo rather than recall responses^20^. CD21^–^CD27^–^ B_m_ cells have also been identified during acute SARS-CoV-2 infection and post-SARS-CoV-2 vaccination^22,25–29^. The S^+^ CD21^–^CD27^–^ B_m_ cells identified here were transcriptionally very similar to their ‘atypical’ counterparts in SLE. These results suggest that CD21^–^CD27^–^ B_m_ cells partake in the normal immune response to pathogens^37^. BCR and IFN-γ signaling appears to be a defining feature of CD21^–^CD27^–^ B_m_ cells, and probably induces and governs the T-bet-dependent transcriptional program in these cells^32^. We found indication of increased BCR and IFN-γ signaling in S^+^ CD21^–^CD27^–^ B_m_ cells, in accord with the increased expression of T-bet and the T-bet target genes *ZEB2* and *ITGAX*^30^.

The heterogeneity of B_m_ cells could be explained by several models^38,39^. The various B_m_ cell subsets could comprise entirely separate lineages, with distinct BCR repertoires. Alternatively, single B cell clones could give rise to different B_m_ cell subsets, with stably imprinted phenotypes or show plasticity. Our longitudinal analysis found that distinct B_m_ cell subsets were clonally related, suggesting plasticity of B_m_ cell subsets. Studies in patients with SLE or HIV infection have suggested that CD21^–^CD27^–^ B_m_ cells differentiate through an extrafollicular pathway^16,17^. We found that the various S^+^ B_m_ cell subsets contained comparable amounts of SHM, suggesting that CD21^–^CD27^–^ B_m_ cells originated either from the GC or from a GC-derived progenitor B_m_ cell upon antigen rechallenge. The latter possibility fits well with our clonal data. It is unclear whether the CD21^–^CD27^–^ B_m_ cells observed post-vaccination can again become resting B_m_ cells or whether this phenotype is terminally fated. Our data showing expression of *ZEB2* in CD21^–^CD27^–^ B_m_ cells suggest unidirectional plasticity, as ZEB2 acts together with T-bet to commit CD8^+^ effector T cells to a terminal differentiation state and has been proposed to act similarly in B cells^16,40^.

Whether CD21^–^CD27^–^ B_m_ cells contribute to protective immunity during infection in humans remains controversial^41^. T-bet^+^ B cells have a protective role in mouse models of acute and chronic viral infections^38,42^. However, antibody responses to several previously applied vaccines were normal in T-bet-deficient patients^30^. CD21^–^CD27^–^ B_m_ cells were reported to be able to secrete antibodies when receiving T cell help and to act as antigen-presenting cells^24^. We found that S^+^ CD21^–^CD27^–^ B_m_ cells showed signs of increased antigen processing and presentation; how much this might translate into truly increased capacity of antigen presentation is unclear^43^.

We found that SARS-CoV-2 infection and vaccination induced long-lived and stable antigen-specific B_m_ cells in the circulation that continued to mature up to 1 year post-infection, as evidenced by their elevated proliferation rate at month 6, high SHM counts and improved breadth of SARS-CoV-2 antigen recognition. This is in line with previous reports that SARS-CoV-2 infection and mRNA vaccination led to lasting B_m_ cell maturation through an ongoing GC reaction^26,44–46^.

These observations in circulating B_m_ cells were paralleled by the appearance of resting B_m_ cells in tonsils, where they showed high expression of CD69 and CD21 and comparable SHM counts to circulating B_m_ cells. CD69 expression is a hallmark of tissue residency in T cells^3^ and has been proposed to characterize resident B_m_ cells in lymphoid and nonlymphoid tissues^47–49^. Phenotype, chemokine receptor expression and clonal connections suggested these cells formed from CD21^+^ resting B_m_ cells, although we cannot exclude that some might have arisen directly in the tonsils.

One limitation of our study is that we performed the clonal analysis after vaccination recall, because the numbers of S^+^ B_m_ cells during acute SARS-CoV-2 infection were too low for our sequencing approach. Moreover, our multimer staining approach might miss low-affinity antigen binders^50^. The inclusion of patients with severe COVID-19 will have increased the average age of our cohort, whereas the individuals from which the tonsil samples were obtained were younger on average.

On the basis of our data, we suggest a linear–plastic model where the antigen stimulation and GC maturation of SARS-CoV-2-specific B cells resulted in the gradual adoption of a CD21^+^Ki-67^lo^ resting B_m_ cell state at months 6–12 post-infection. These circulating resting B_m_ cells might be able to rapidly respond to antigen rechallenge with the acquisition of different B_m_ cell fates or they might home to secondary lymphoid and peripheral organs to form a CD69^+^ tissue-resident B_m_ cells. Our work also provides insight into the CD21^–^CD27^–^ B_m_ cells, which made up a sizeable portion of B_m_ cells following acute viral infection and vaccination in humans.

## Methods

### Patient cohorts

This study was approved by the Cantonal Ethics Committee of Zurich (BASEC #2016-01440). Patients with COVID-19 and healthy individuals were recruited at one of four hospitals in the Canton of Zurich, Switzerland. All study participants provided written informed consent. Serum and blood was obtained, and peripheral blood mononuclear cells were isolated by density centrifugation, washed and frozen in fetal bovine serum (FBS) with 10% dimethyl sulfoxide and stored in liquid nitrogen until use.

A longitudinal cohort (Extended Data Fig. 1a and Supplementary Table 1) consisted of individuals with reverse-transcriptase polymerase chain reaction-confirmed, symptomatic SARS-CoV-2 infection at acute infection (April to September 2020) and months 6 and 12 after infection, including patients with mild (*n* = 42) and severe (*n* = 23) COVID-19. Of these, 35 received SARS-CoV-2 mRNA vaccination between month 6 and month 12, and 3 subjects between acute infection and month 6. We included a total of 65 patients of the full cohort^51,52^ on the basis of a power calculation from pre-experiments and according to sample availability of at least paired samples from two timepoints. The flow cytometry and scRNA-seq subcohort characteristics are presented in Supplementary Tables 1 and 2, respectively.

Another cohort (Extended Data Fig. 1b and Supplementary Table 3) comprised subjects seen at University Hospital Zurich between November 2021 and April 2022 that underwent tonsillectomy for recurrent and chronic tonsillitis or obstructive sleep apnea and were exposed to SARS-CoV-2 by infection and/or vaccination. If they had a confirmed SARS-CoV-2 infection and/or SARS-CoV-2 nucleocapsid-specific antibodies, they were considered ‘SARS-CoV-2-recovered’. The cohort size was based on sample availability. We obtained paired tonsil and peripheral blood mononuclear cell and serum samples. Tonsils were processed according to established protocols^47,53^. Briefly, they were cut into small pieces, ground through 70 μm cell strainers, and washed in phosphate-buffered saline (PBS), before performing density gradient centrifugation. Subsequently, the mononuclear cells were frozen in FBS with 10% dimethyl sulfoxide and stored in liquid nitrogen until use.

We recruited 11 healthy controls (Extended Data Fig. 1c and Supplementary Table 4) with no history of SARS-CoV-2 infection and seronegative for SARS-CoV-2 S S1-specific antibodies. They donated blood before vaccination, at days 8–13 (week 2) post-second dose, 6 months after the second dose and days 11–14 post-third dose. All individuals received the Pfizer/BioNTech (BNT162b2) mRNA vaccine.

### Spectral flow cytometry

To stain antigen-specific B cells, biotinylated SARS-CoV-2 S, RBD, nucleocapsid (MiltenyiBiotec) and H1N1 (A/California/07/2009, SinoBiological) were incubated individually with fluorescently labeled SAV at 4:1 molar ratio for SARS-CoV-2 proteins and 6:1 for influenza antigen, with SAV added stepwise every 15 min at 4 °C for 1 h (refs. ^22,54^). The probes were mixed in 1:1 Brilliant Buffer (BD Bioscience) and FACS buffer (PBS with 2% FBS and 2 mM EDTA) with 5 μM of free d-biotin. We stained S, RBD, nucleocapsid (for tonsil samples), hemagglutinin (for tonsil samples) or a decoy probe using separate fluorochrome-conjugated SAVs. Frozen mononuclear cells were stained in 96-well U-bottom plates using ZombieUV Live-Dead staining (BioLegend) and TruStain FcX (1:200, BioLegend) in PBS for 30 min, followed by staining with the above-mentioned antigen-specific staining mix (200 ng S, 50 ng RBD, 100 ng nucleocapsid, 100 ng hemagglutinin and 20 ng SAV-decoy per color per 50 μl) at 4 °C for 1 h. Subsequently, cells were stained for 30 min with surface markers, followed by fixation and permeabilization with transcription factor staining buffer (eBioscience) at room temperature for 1 h and intracellular staining at room temperature for 30 min, before washing and acquisition. The antibodies used are listed in Supplementary Tables 5 and 7. Samples were acquired on a Cytek Aurora cytometer using the SpectroFlo software. The same positive control from a SARS-CoV-2-vaccinated healthy control was included in every experiment to ensure consistent results.

### Flow cytometry analysis

Flow cytometry data were analyzed with FlowJo (version 10.8.0), with gating strategies shown in Extended Data Figs. 2 and 5. Subsets and markers of antigen-specific B cells and antigen-specific B cell subsets were evaluated only if more than nine or three specific cells per sample were detected, respectively. Dimensionality reduction and clustering analysis of flow cytometry data were performed in R using the CATALYST workflow (CATALYST package, version 1.18.1) (ref. ^55^). Markers were scaled with arcsinh transformation (cofactor 6,000), samples were subsetted to maximally 25 S^+^ B_m_ cells per sample. For UMAP representations and PhenoGraph clustering (Rphenograph package, version 0.99.1) (ref. ^56^), with *k* set to 20, the following B cell markers were used: CD11c, CD19, CD20, CD21, CD24, CD27, CD38, CD71, CD80, CXCR5, BAFF-R, FcRL5, IgA, IgD, IgG, IgM, Blimp1, IRF8, Ki67 and Tbet.

### SARS-CoV-2-specific antibody measurement

Anti-SARS-CoV-2 antibodies were measured by a commercially available enzyme-linked immunosorbent assay specific for S1 of SARS-CoV-2 (Euroimmun SARS-CoV-2 IgG and IgA)^57^ or by a bead-based multiplexed immunoassay^58^.

### Cell sorting for scRNA-seq, scRNA-seq and library preparation

scRNA-seq was performed on samples from nine patients of the SARS-CoV-2 Infection Cohort (Supplementary Table 2), three of the SARS-CoV-2 Vaccination Cohort, and paired blood and tonsil samples of four patients of the SARS-CoV-2 Tonsil Cohort (two recovered and two only vaccinated). Samples were stained as described for spectral flow cytometry using biotinylated S^WT^, RBD, S^beta^ and S^delta^ (MiltenyiBiotec) and hemagglutinin (SinoBiological) that were multimerized at 4:1 molar ratios with fluorescently labeled and/or barcoded SAV (TotalSeqC, BioLegend). Following 20 min staining with fixable viability dye eFluor 780 (eBioscience) and TruStain FcX and subsequently 1 h antigen-specific staining mix, cells were incubated at 4 °C for 30 min with a surface staining mix containing fluorescently labeled and barcoded antibodies, and each sample was marked with a hashtag antibody that allowed multiplexing (Supplementary Table 6). Cells were sorted on a FACS Aria III 4L sorter using the FACS Diva software. Antigen-specific cells per sample were sorted with 1,500–2,000 nonspecific B cells, as shown in Extended Data Figs. 2d and 6a. Sorted B cells were analyzed by scRNA-seq using the commercial 5′ Single Cell GEX and VDJ v1.1 platform (10x Genomics). After sorting, cell suspensions were pelleted at 400*g* for 10 min at 4 °C, resuspended and loaded into the Chromium Chip following the manufacturer’s instructions. Fourteen cycles (in one case 17) of initial cDNA amplification were used for all sample batches, and single-cell sequencing libraries for whole-transcriptome analysis (GEX), BCR profiling (VDJ) and TotalSeq (BioLegend) barcode detection (ADT) were generated. Final libraries were quantified using a Qubit Fluorometer, pooled at ratios of 5:1:1 or 10:1:1 (GEX:VDJ:ADT) and sequenced on a NovaSeq 6000 system.

### Single-cell transcriptome analysis

Preprocessing of raw scRNA-seq data was done as described^51^. Briefly, FASTQ files were aligned to the human GRCh38 genome using Cell Ranger’s ‘cellranger multi’ pipeline (10x Genomics, v6.1.2) with default settings, which allowed one to process together the paired GEX, ADT and VDJ libraries for each sample batch. Downstream analysis was conducted in R version 4.1.0 mainly with the package Seurat (v4.1.1) (ref. ^59^). In the SARS-CoV-2 Infection Cohort, cells with fewer than 200 or more than 2,500 detected genes and cells with more than 10% detected mitochondrial genes were excluded from the analysis. In the SARS-CoV-2 Tonsil Cohort and SARS-CoV-2 Vaccination Cohort, cells with fewer than 200 or more than 4,000 detected genes were excluded from the analysis. For the SARS-CoV-2 Tonsil Cohort, we used a cutoff of 7.5% detected mitochondrial genes. Gene expression levels were log normalized using Seurat’s NormalizeData() function with default settings. Sample assignment of cells was done using TotalSeq-based cell hashing and Seurat’s HTODemux() function. When comparing dataset quality, we noticed a markedly lower median gene detection and unique molecular identifier count per cell in one of our datasets of the SARS-CoV-2 Infection Cohort. We associated this with an incident during sample preparation in one of our experiments and decided to exclude most cells of this dataset from the analysis.

As an internal reference for SHM counts in naïve B cells, we co-sorted naïve B cells in one experiment of the SARS-CoV-2 Infection Cohort. Naïve B cell clusters were identified on the basis of their surface protein expression of CD27, CD21 and IgD and their transcriptional levels of *TCLA1*, *IL4R*, *BACH2*, *IGHD* and *BTG1*. Independent datasets were then integrated using Seurat’s anchoring-based integration method. Gene expression data and TotalSeq surface proteome data were integrated separately. Seurat’s WNN analysis was used to take advantage of our multimodal approach during clustering and visualization^59^. Clustering was performed using the Louvain algorithm and a resolution of 0.4. For UMAP generation in the SARS-CoV-2 Infection Cohort datasets, the embedding parameters were manually set to *a* = 1.4 and *b* = 0.75. Differential gene expression analyses were done using assay ‘RNA’ of the integrated datasets. FindAllMarkers and FindMarkers functions were executed with logfc.thresholds set to 0.25 (0.1 for comparing resting B_m_ cells at month 6 versus month 12) and a min.pct cutoff at 0.1. Heat maps were generated using the ComplexHeatmap package (v2.13.1) or pheatmap package (v1.0.12) (ref. ^60^).

Gene set enrichment analysis (GSEA) was done as described^51^. Briefly, lists of differentially expressed genes were preranked in decreasing order by the negative logarithm of their *P* value, multiplied for the sign of their average log-fold change (in R, ‘-log(P_val)*sign(avg_log2FC)’). GSEA was performed on this preranked list using the R package fgsea (v.1.2). Gene sets were obtained from the Molecular Signatures Database (v7.5.1, collections H and C5) and loaded in R by the package msigdbr (v.7.5.1). To make the results reproducible, seed value was set (‘set.seed(42)’ in R) before execution. A multiple hypothesis correction procedure was applied to obtain adjusted *P* values. Results were filtered for gene sets that were significantly enriched with adjusted *P* < 0.05.

Gene set variation analysis with the package gsva (v1.42.0) was used to estimate gene set enrichments for more than two groups^61^. Transcriptomes of individual cells were used as inputs for the gsva() function with default parameters. Gene set enrichments for individual cells were summarized to patient pseudobulks by calculating mean enrichment values of cells belonging to the same patient. Pseudobulking was done only for patients with more than 20 cells in each cell subset. Resulting scores were used to compute fold changes and significance levels for enrichment score comparisons between cell subsets in limma (v3.50.3) (ref. ^62^).

Single-cell trajectories were created with Monocle3 (version 1.2.9) (ref. ^63^). Raw counts obtained from the cellranger gene expression matrix were used to create cell datasets, which were preprocessed using the Monocle 3 pipeline. Different batches were aligned using Batchelor (v.1.10.0) (ref. ^64^). The num_dim parameter of Monocle’s preprocess_cds() function was set to 20. Functions reduce_dimension(), order_cells() and graph_test() were executed with default parameters.

### BCR analysis

B cell clonality analysis was performed mainly with the changeo-10x pipeline from the Immcantation suite^65^ using the singularity image provided by Immcantation developers. filtered_contig_annotations.csv files obtained from the cellranger multipipeline were used as input for the changeo-10x pipeline. Unique combinations of bases were appended to cell barcodes per batch before combining the data from different batches of sequencing to prevent cell barcode collisions. The clonality distance threshold was set to 0.20 for the longitudinal analysis of the SARS-CoV-2 Infection Cohort dataset and to 0.05 for the SARS-CoV-2 Tonsil Cohort dataset. Visualization of the clonal trees was done using dowser^66^. BCR variable gene segment usage was additionally quantified using the R package scRepertoire (v.1.3.5) (ref. ^67^). Clonal diversity between B_m_ cell subsets was investigated using the alphaDiversity function of Immcantations package Alakazam (v1.2.0) (ref. ^65^). Segment usage between B_m_ cell subsets was compared using edgeR (v3.36). For this, a count matrix was created with HC/LC segments as rows and samples as columns. Standard edgeR workflow was used to create a linear model for the count data and to conduct statistical tests for differential segment usage between B_m_ cell subsets.

### Mapping of BCR sequences to antigen specificity

We used an adaptation of LIBRA-seq^68^ to identify antigen-specific cells in our sequencing data. Following subtraction of raw counts of baiting-negative control from those of all other antigen-baiting constructs in every cell, cutoffs for background binding levels were manually determined for every construct by inspection of bimodal distributions of count frequencies across all cells, and all binding counts below thresholds were set to zero and classified as nonbinding. Seurat’s centered log ratio transformation was applied across features, followed by a scaling of obtained values, resulting in final LIBRA scores. Cells with LIBRA scores >0 for the respective antigens were defined as antigen-specific, and in the SARS-CoV-2 infection, cohort cells were considered S^+^ if any of the antigens used for baiting (S^WT^, S^beta^, S^delta^, RBD) were defined as specific.

### Statistical analysis

The number of samples and subjects and the statistical tests used in each experiment are indicated in the corresponding figure legends. All tests were performed two-sided. We did not assume normal distribution for the flow cytometry data and used nonparametric tests such as Kruskal–Wallis to test for differences between continuous variables in more than two groups, and *P* values were adjusted for multiple testing using Dunn’s method. For scRNA-seq data, distribution was assumed to be normal, but this was not formally tested. Statistical analysis was performed with GraphPad Prism (version 9.4.1, GraphPad Software, USA) and R (version 4.1.0). Statistical significance was established at *P* < 0.05.

### Reporting summary

Further information on research design is available in the Nature Portfolio Reporting Summary linked to this article.

## Online content

Any methods, additional references, Nature Portfolio reporting summaries, source data, extended data, supplementary information, acknowledgements, peer review information; details of author contributions and competing interests; and statements of data and code availability are available at 10.1038/s41590-023-01497-y.

## Supplementary information

Supplementary Information Supplementary Tables 1–7.

Reporting Summary

Peer Review File

## Data Availability

The sequencing data have been deposited at Zenodo at 10.5281/zenodo.7064118. The flow cytometry dataset is available upon request from the corresponding authors. Source data are provided with this paper.

## Source data

Source Data Fig. 1 Statistical source data.
Source Data Fig. 2 Statistical source data.
Source Data Fig. 3 Statistical source data.
Source Data Fig. 4 Statistical source data.
Source Data Fig. 5 Statistical source data.
Source Data Fig. 6 Statistical source data.
Source Data Extended Data Fig. 2 Statistical source data.
Source Data Extended Data Fig. 4 Statistical source data.
Source Data Extended Data Fig. 5 Statistical source data.
Source Data Extended Data Fig. 7 Statistical source data.
Source Data Extended Data Fig. 8 Statistical source data.

## Code availability

The code generated during the current study is available at https://github.com/Moors-Code/MBC_Plasticity_Moor_Boyman_Collaboration.
