# Publisher Correction: Human memory B cells show plasticity and adopt multiple fates upon recall response to SARS-CoV-2

**DOI:** 10.1038/s41590-023-01628-5

**Published:** 2023-08-21

**Authors:** Yves Zurbuchen, Jan Michler, Patrick Taeschler, Sarah Adamo, Carlo Cervia, Miro E. Raeber, Ilhan E. Acar, Jakob Nilsson, Klaus Warnatz, Michael B. Soyka, Andreas E. Moor, Onur Boyman

**Affiliations:** 1 Department of Immunology, University Hospital Zurich, Zurich, Switzerland; 2 Department of Biosystems Science and Engineering, ETH Zurich, Basel, Switzerland; 3 Department of Rheumatology and Clinical Immunology, Faculty of Medicine, University of Freiburg, Freiburg, Germany; 4 Center for Chronic Immunodeficiency, Faculty of Medicine, University of Freiburg, Freiburg, Germany; 5 Department of Otorhinolaryngology, Head and Neck Surgery, University and University Hospital Zurich, Zurich, Switzerland; 6 Faculty of Medicine and Faculty of Science, University of Zurich, Zurich, Switzerland

**Keywords:** Immunological memory, Viral infection, Tonsils, Humoral immunity

Correction to: *Nature Immunology* 10.1038/s41590-023-01497-y, published 27 April 2023.

In the version of this article initially published, there was a typo in the *y*-axis in the Fig. 4f graph, where “CD27_APC-Cy7_” originally read “CD21_APC-Cy7_”. This has been corrected in the HTML and PDF versions of the article.

