# Supplementary material for: Human memory B cells show plasticity and adopt multiple fates upon recall response to SARS-CoV-2
Source: Nat Immunol. 2023 Apr 27;24(6):955–65. doi: 10.1038/s41590-023-01497-y (PMC10232369; doi:10.1038/s41590-023-01497-y)
Supplement: Supplementary file 1 — Supplementary Tables 1–7. [file 41590_2023_1497_MOESM1_ESM.pdf]

nature immunology

Article

https://doi.org/10.1038/s41590-023-01497-y

Human memory B cells show plasticity and adopt multiple fates upon recall response to SARS-CoV-2

In the format provided by the authors and unedited

Supplementary Table 1. Characteristics of SARS-CoV-2 Infection Cohort.

**Time point of sampling**		**Acute infection (n = 59)**	**Month 6 Post-infection (n = 64)**		**Month 12 Post-infection (n = 55)**	
			Not vaccinated (n = 61)	Vaccinated (n = 3)	Not vaccinated (n = 17)	Vaccinated (n = 38)
Age, y median (IQR)		39 (32 – 58)	41 (32.5 – 59.5)	68 (22 – 69)	39 (30.5 – 60)	39.5 (32 – 56.5)
Sex, female/male		30/29	30/31	3/0	7/10	18/20
**Severities**						
Mild	Mild illness, no.	37	36	2	11	27
	Pneumonia, no.	3	3	1	–	3
Severe	Severe pneumonia, no.	6	9	–	2	4
	Mild ARDS, no.	1	2	–	1	1
	Moderate ARDS, no.	5	5	–	2	1
	Severe ARDS, no.	7	6	–	1	2
Days post symptom onset, mean (±SD)		13.71 (±9.51)	201.9 (±28.94)	208.7 (±29.67)	374.2 (±23.44)	377.6 (±22.5)
**SARS-CoV-2 vaccinations**[a]						
Moderna, no. (1/2 doses)		–	–	1/0	–	20/3
BioNTech, no. (1/2 doses)		–	–	0/2	–	7/7
Days post vaccination, mean (±SD)		–	–	16 (±6.25)	–	72.82 (±48.35)
**SARS-CoV-2-specific antibodies**						
Spike S1 IgA, OD ratio mean (±SD)		4.53 (±4.02)	3.65 (±2.51)	8.36 (±0.38)	3.45 (±2.91)	8.97 (±1.45)
Spike S1 IgG, OD ratio mean (±SD)		3.09 (±3.77)	4.28 (±3.01)	11.24 (±0.68)	4.0 (±3.48)	10.0 (±1.22)

Abbreviations: IQR, interquartile range; OD, optical density; SD, standard deviation.
[a] The information on which SARS-CoV-2 mRNA vaccine was applied was not retrievable in one patient.

Supplementary Table 2. Characteristics of single-cell RNA sequencing subcohort of SARS-CoV-2 Infection Cohort.

Patient ID	Sex	Timepoint	Age [y]	COVID-19 Severity	Days post symptom onset	Vaccination	Days post vaccination
CovSeq_P1	Female	Month 6	59	Mild	214	Not vaccinated	–
		Month 12	60	Mild	408	Not vaccinated	–
CovSeq_P2	Female	Month 6	69	Sev ARDS	117	Not vaccinated	–
		Month 12	70	Sev ARDS	312	Not vaccinated	–
CovSeq_P3	Male	Month 6	59	Mod ARDS	255	Not vaccinated	–
		Month 12	59	Mod ARDS	394	Not vaccinated	–
CovSeq_P4	Female	Month 6	42	Mild	181	Not vaccinated	–
		Month 12	42	Mild	377	Moderna, 1 dose	9
CovSeq_P5	Male	Month 6	53	Mild	177	Not vaccinated	–
		Month 12	53	Mild	372	Moderna, 1 dose	11
CovSeq_P6	Male	Month 6	76	Sev Pneumonia	203	Not vaccinated	–
		Month 12	77	Sev Pneumonia	384	Moderna, 1 dose	23
CovSeq_P7	Female	Month 6	32	Mild	189	Not vaccinated	–
		Month 12	32	Mild	364	Moderna, 1 dose	85
CovSeq_P8	Female	Month 6	54	Mild	282	Not vaccinated	–
		Month 12	54	Mild	415	Moderna, 1 dose	87
CovSeq_P9	Male	Month 6	27	Mild	193	Not vaccinated	–
		Month 12	27	Mild	377	Moderna, 1 dose	108

Supplementary Table 3. Characteristics of SARS-CoV-2 Tonsil Cohort.

	**Vaccinated (n = 8)**	**Recovered (n = 8)**
Age, y median (IQR)	33 (25.5 – 43.5)	25 (22 – 37)
Sex, female/male	2/6	7/1
Anamnestic COVID-19, no.	–	6
**SARS-CoV-2 vaccinations**		
Vaccine doses, no. (0/1/2/3)	0/0/6/2	2/1/2/3
Vaccine, no. (Moderna/BioNTech/ combined mRNA/unknown)	6/0/0/2	1/4/1/0
Days after last vaccination, mean (±SD)	144.1 (±51.61)	117.5 (±56.53)
**SARS-CoV-2-specific antibodies**		
Spike S1 IgG (SOC), mean (±SD)	131.5 (±94.54)	177.9 (±98.08)
Spike S2 IgG (SOC), mean (±SD)	2.4 (±1.21)	5.17 (±2.76)
RBD IgG (SOC), mean (±SD)	107.8 (±62.51)	134.0 (±73.29)
N IgG (SOC), mean (±SD)	0.16 (±0.03)	22.41 (±29.12)
Abbreviations: IQR, interquartile range; N, nucleocapsid; OD, optical density; RBD, receptor-binding domain; SD, standard deviation; SOC, signal-over cut-off.		

Supplementary Table 4. Characteristics of SARS-CoV-2 Vaccination Cohort.

	Baseline (n = 10)	Week 2 post-second dose (n = 10)	Month 6 post-second dose (n = 11)	Week 2 post-third dose (n = 11)
Age, y median (IQR)	29.5 (28 – 32)	29.5 (28 – 32)	29 (28 – 32)	29.50 (27.5 – 32)
Sex, female/male	4/6	4/6	5/6	5/5
**SARS-CoV-2 vaccinations**				
Vaccine (Moderna/ BioNTech)	–	0/10	0/11	0/10
Days after first dose, mean (±SD)	–	39.9 (±1.52)	243.8 (±24.57)	269.4 (±23.31)
Days after third dose, mean (±SD)	–	–	–	11.7 (±1.06)
**SARS-CoV-2-specific antibodies**				
Spike S1 IgA, OD ratio mean (±SD)	0.38 (±0.21)	8.87 (±0.2)	3.66 (±3.46)	8.81 (±1.3)
Spike S1 IgG, OD ratio mean (±SD)	0.12 (±0.03)	11.01 (±0.64)	7.05 (±1.21)	>10 (±0.0)

Abbreviations: IQR, interquartile range; OD, optical density; SD, standard deviation.

Supplementary Table 5. Antibodies used for B cell staining.

Antigen	Fluorophore	Provider	Dilution	Cat No
CD3	SparkBlue550	Biolegend	1:200	344852
CD11c	BUV615	BD Biosciences	1:200	612967
CD14	SparkBlue550	Biolegend	1:400	367147
CD19	SparkNIR 685	Biolegend	1:100	302270
CD20	BUV563	BD Biosciences	1:400	748456
CD21	BUV496	BD Biosciences	1:200	750614
CD24	BUV805	BD Biosciences	1:200	742010
CD27	APC-Cy7	Biolegend	1:200	356424
CD38	APC-Fire810	Biolegend	1:200	303549
CD71	PerCP-Cy5.5	Biolegend	1:100	334114
CD80	PE-Cy5	Biolegend	1:100	305209
CXCR5	BV750	Biolegend	1:100	356941
BAFF-R	BV605	BD Biosciences	1:200	743571
FcRL5	BUV615	BD Biosciences	1:100	751131
IgD	BV480	BD Biosciences	1:400	566187
IgM	BV570	Biolegend	1:100	314517
IgA	APC	Miltenyi Biotec	1:400	130-113-472
IgG	BUV737	BD Biosciences	1:400	741858
IgG1	PE	Cytognos	1:200	CYT-IGG1PE
IgG3	FITC	Cytognos	1:200	CYT-IGG3F
BLIMP1	PE-Dazzle594	BD Biosciences	1:100	565274
IRF8	V450	Invitrogen	1:100	48-9852-82
Ki67	BUV395	BD Biosciences	1:100	564071
Tbet	BV711	Biolegend	1:100	644819
ZombieUV		Biolegend	1:400	423107
Streptavidin	BV421	Biolegend	see Methods	405226
Streptavidin	BV650	Biolegend	see Methods	405231
Streptavidin	BV785	Biolegend	see Methods	405249
Streptavidin	PE-Cy7	Biolegend	see Methods	405206

Supplementary Table 6. Antibodies used for fluorescently-activated cell sorting.

Antigen	Cohort	Fluorophore	Provider	Dilution	Cat No
CD3	COVID-19, Tonsil, Vac	BV510	Biolegend	1:100	317332
CD14	COVID-19, Tonsil, Vac	BV510	Biolegend	1:100	301841
CD19	COVID-19, Tonsil, Vac	FITC	Biolegend	1:100	302206
CD21	COVID-19, Tonsil, Vac	TotalSeq™-C0181	Biolegend	1:2000	354923
CD27	COVID-19, Tonsil, Vac	PE-Dazzle	Biolegend	1:200	356421
CD27	COVID-19, Tonsil, Vac	TotalSeq™-C0154	Biolegend	1:715	302853
CD38	COVID-19, Tonsil, Vac	PE-Cy7	Biolegend	1:150	303515
CD38	Tonsil	TotalSeq™-C0410	Biolegend	1:1000	356637
CD45RB	Tonsil	TotalSeq™-C0844	Biolegend	1:2000	310211
CD62L	Tonsil	TotalSeq™-C0147	Biolegend	1:2000	304851
CD69	Tonsil	TotalSeq™-C0146	Biolegend	1:500	310951
CD71	COVID-19, Tonsil, Vac	TotalSeq™-C0394	Biolegend	1:1000	334125
CXCR3	Tonsil	TotalSeq™-C0140	Biolegend	1:500	353747
CXCR5	COVID-19, Tonsil, Vac	TotalSeq™-C0144	Biolegend	1:500	356939
CCR6	Tonsil	TotalSeq™-C0143	Biolegend	1:1000	353440
FcRL5	COVID-19, Tonsil, Vac	TotalSeq™-C0829	Biolegend	1:500	340309
IgD	COVID-19, Tonsil, Vac	Alexa647	Biolegend	1:200	348227
IgD[a]	COVID-19, Tonsil, Vac	TotalSeq™-C0384	Biolegend	1:500	348245
Hashtag 1	COVID-19, Tonsil, Vac	TotalSeq™-C0251	Biolegend	1:50	394661
Hashtag 2	COVID-19, Tonsil, Vac	TotalSeq™-C0252	Biolegend	1:50	394663
Hashtag 3	COVID-19, Tonsil, Vac	TotalSeq™-C0253	Biolegend	1:50	394665
Hashtag 4	COVID-19, Tonsil, Vac	TotalSeq™-C0254	Biolegend	1:50	394667
Hashtag 5	COVID-19, Tonsil, Vac	TotalSeq™-C0255	Biolegend	1:50	394669
Hashtag 6	COVID-19, Tonsil, Vac	TotalSeq™-C0256	Biolegend	1:50	394671
Hashtag 7	COVID-19, Tonsil	TotalSeq™-C0257	Biolegend	1:50	394673
Hashtag 8	COVID-19, Tonsil	TotalSeq™-C0258	Biolegend	1:50	394675
Hashtag 9	COVID-19	TotalSeq™-C0259	Biolegend	1:50	394677
Hashtag 10	COVID-19	TotalSeq™-C0260	Biolegend	1:50	394679
Streptavidin	COVID-19, Tonsil, Vac	TotalSeq™-C0951 PE	Biolegend	see Methods	405261
Streptavidin	COVID-19, Tonsil, Vac	TotalSeq™-C0952 PE	Biolegend	see Methods	405263
Streptavidin	COVID-19, Vac	TotalSeq™-C0954 PE	Biolegend	see Methods	405267
Streptavidin	COVID-19, Tonsil, Vac	TotalSeq™-C0971	Biolegend	see Methods	405271
Streptavidin	COVID-19, Tonsil, Vac	TotalSeq™-C0972	Biolegend	see Methods	405273

Streptavidin	Tonsil, Vac	TotalSeq™-C0973	Biolegend	see Methods	405275
Streptavidin	Vac	TotalSeq™-C0974	Biolegend	see Methods	405277
Streptavidin	COVID-19, Tonsil, Vac	BV421	Biolegend	see Methods	405226
Streptavidin	COVID-19, Tonsil, Vac	BV785	Biolegend	see Methods	405249
Fixable Viability Dye	COVID-19, Tonsil, Vac	eFluor™ 780	Invitrogen	1:1000	65-0865-14
[a] For the SARS-CoV-2 infection cohort only used in the sample set where naïve B cells were sorted.					

Supplementary Table 7. Antibodies used for staining of tonsillar samples.

Antigen	Panels	Fluorophore	Provider	Dilution	Cat No
CD3	1, 2	BV510	Biolegend	1:100	317332
CD14	1	SparkBlue550	Biolegend	1:400	367147
CD14	2	BV510	Biolegend	1:200	301851
CD19	1	SparkNIR 685	Biolegend	1:100	302270
CD19	2	FITC	Biolegend	1:100	302206
CD20	1, 2	BUV563	BD Biosciences	1:400	748456
CD21	1, 2	BUV496	BD Biosciences	1:200	750614
CD27	1, 2	APC-Cy7	Biolegend	1:200	356424
CD38	1, 2	APC-Fire810	Biolegend	1:200	303549
CD45RB	1	APC	Invitrogen	1:100	MA1-19461
CD45RB	2	PE	Biolegend	1:100	310204
CD56	2	BV510	Biolegend	1:100	318339
CD69	1	BV605	Biolegend	1:50	310937
CD69	2	PE-Dazzle594	Biolegend	1:100	310941
CXCR3	2	BV605	Biolegend	1:100	353729
CXCR4	1, 2	PE-Cy5	Biolegend	1:100	306507
CXCR5	1, 2	BV750	Biolegend	1:50	356941
FcRL4	2	APC	Biolegend	1:100	340205
FcRL5	1, 2	BUV615	BD Biosciences	1:100	751131
IgD	1, 2	BV480	BD Biosciences	1:400	566187
IgM	1, 2	BV570	Biolegend	1:100	314517
IgG	1, 2	BUV805	BD Biosciences	1:800	742041
IgG1	1	PE	Cytognos	1:200	CYT-IGG1PE
IgA	1, 2	PerCP-Vio700	Miltenyi Biotec	1:400	130-114-004
BCL6	1	PE-Cy7	Biolegend	1:100	358511
BLIMP1	1	PE-Dazzle594	BD Biosciences	1:100	565274
Ki67	1, 2	BUV395	BD Biosciences	1:100	564071
Tbet	1, 2	BV711	Biolegend	1:100	644819
ZombieUV	1, 2		Biolegend	1:400	423107
Streptavidin	1, 2	BUV661	BD Biosciences	see Methods	612979
Streptavidin	1, 2	BUV737	BD Biosciences	see Methods	612775
Streptavidin	1, 2	BV421	Biolegend	see Methods	405226
Streptavidin	1, 2	BV650	Biolegend	see Methods	405231
Streptavidin	1, 2	BV785	Biolegend	see Methods	405249
Streptavidin	1	KIRAVIA Blue 520	Biolegend	see Methods	405171
