# Supplementary material for: Human memory B cells show plasticity and adopt multiple fates upon recall response to SARS-CoV-2
Source: Nat Immunol. 2023 Apr 27;24(6):955–65. doi: 10.1038/s41590-023-01497-y (PMC10232369; doi:10.1038/s41590-023-01497-y)
Supplement: Supplementary file 2 — Reporting Summary [file 41590_2023_1497_MOESM2_ESM.pdf]

nature portfolio

Corresponding author(s): Onur Boyman, Andreas E. Moor

Last updated by author(s): Mar 20, 2023

Reporting Summary

Nature Portfolio wishes to improve the reproducibility of the work that we publish. This form provides structure for consistency and transparency in reporting. For further information on Nature Portfolio policies, see our Editorial Policies and the Editorial Policy Checklist.

Statistics

For all statistical analyses, confirm that the following items are present in the figure legend, table legend, main text, or Methods section.

n/a	Confirmed	
☐	☒	The exact sample size (n) for each experimental group/condition, given as a discrete number and unit of measurement
☐	☒	A statement on whether measurements were taken from distinct samples or whether the same sample was measured repeatedly
☐	☒	The statistical test(s) used AND whether they are one- or two-sided<br>*Only common tests should be described solely by name; describe more complex techniques in the Methods section.*
☒	☐	A description of all covariates tested
☐	☒	A description of any assumptions or corrections, such as tests of normality and adjustment for multiple comparisons
☐	☒	A full description of the statistical parameters including central tendency (e.g. means) or other basic estimates (e.g. regression coefficient) AND variation (e.g. standard deviation) or associated estimates of uncertainty (e.g. confidence intervals)
☐	☒	For null hypothesis testing, the test statistic (e.g. F, t, r) with confidence intervals, effect sizes, degrees of freedom and P value noted<br>*Give P values as exact values whenever suitable.*
☒	☐	For Bayesian analysis, information on the choice of priors and Markov chain Monte Carlo settings
☒	☐	For hierarchical and complex designs, identification of the appropriate level for tests and full reporting of outcomes
☒	☐	Estimates of effect sizes (e.g. Cohen's d, Pearson's r), indicating how they were calculated

Our web collection on statistics for biologists contains articles on many of the points above.

Software and code

Policy information about availability of computer code

Data collection	Flow cytometry data was generated using Cytek SpectroFlo (Version 3.0.3) and for sorting using BD FACSDiva (Version 8.0.1).
Data analysis	For data analysis Graph-Pad Prism (Version 9.4.1, GraphPad Software, La Jolla California USA) and R (Version 4.1.0) were used. Flow cytometry data were analysed using FlowJo (version 10.8.0), and unsupervised analysis was performed using the CATALYST package (version 1.18.1) and Rphenograph package (version 0.99.1).<br>For the scRNA-seq analysis the following packages were used: Cell Ranger's 'cellranger multi' pipeline (10x Genomics) (Version 6.1.2), Seurat (Version 4.1.1), ComplexHeatmap package (Version 2.13.1), pheatmap package (Version 1.0.12), package fgsea (Version 1.2), package msigdbr (Version 7.5.1), gsva (Version 1.42.0), limma (Version 3.50.3), Monocle3 (Version 1.2.9), Batchelor (Version 1.10.0), changeo-10x pipeline from the immcantation_suite-4.3.0, package scRepertoire (Version 1.3.5), Alakazam (Version 1.2.0), edgeR (Version 3.36). The code generated during the current study is available at https://github.com/Moors-Code/MBC_Plasticity_Moor_Boyman_Collaboration.

For manuscripts utilizing custom algorithms or software that are central to the research but not yet described in published literature, software must be made available to editors and reviewers. We strongly encourage code deposition in a community repository (e.g. GitHub). See the Nature Portfolio guidelines for submitting code & software for further information.

Data

Policy information about availability of data

All manuscripts must include a data availability statement. This statement should provide the following information, where applicable:

- Accession codes, unique identifiers, or web links for publicly available datasets
- A description of any restrictions on data availability
- For clinical datasets or third party data, please ensure that the statement adheres to our policy

The sequencing data has been deposited at zenodo.org and is available under 10.5281/zenodo.7064118. Gene sets were obtained from the Molecular Signatures Database (v7.5.1, collections H and C5). The source data are provided with the article. The flow cytometry dataset is available upon request from the corresponding authors.

Human research participants

Policy information about studies involving human research participants and Sex and Gender in Research.

Reporting on sex and gender	The SARS-CoV-2 infection cohort for the flow cytometry analysis consisted of 33 female and 32 male patients, the tonsil cohort of 9 female and 7 male patients and the vaccination cohort of 5 female and 6 male individuals. Sex was collected from the electronic medical records, gender data was not collected.
Population characteristics	The cohort characterstics for the different (sub-)cohorts are shown in supplementary tables 1-4.
Recruitment	Patients at four hospitals in the Canton of Zurich, Switzerland, that had a reverse-transcriptase polymerase chain reaction confirmed SARS-CoV-2 infection and were symptomatic, were approached whether they would be interested in participating in the study. Patients had to be over 18 years old and had to be competent at the time of consent. Following written informed consent the COVID-19 patients donated blood and serum samples. Subsequently, patients visited again at month 6 and 12 post-infection and donated blood and serum samples at the respective time points. The patients were included in the study during their acute disease between April 2020 and September 2020 and for the 12 months follow-up between April 2021 and September 2021. As the patients had to be competent when providing the informed consent this might have skewed the disease severity distribution of the cohort.<br>Additionally, patients that underwent a tonsillectomy at University Hospital Zurich between November 2021 and April 2022 were approached whether they wold be interested in participating in the study. All patients signed a written informed consent before sample collection. Subsequently paired tonsil and peripheral blood samples, as well as serum samples, were collected. Patients underwent their tonsillectomy for recurrent and chronic tonsillitis or obstructive sleep apnea.<br>We recruited 11 healthy controls that had no history of SARS-CoV-2 infection. After providing a written informed consent the individuals donated blood before the vaccination, 8-13 days after the second vaccine shot, six months after the vaccination as well as 11-14 days after the third vaccine dose. All donors were seronegative for SARS-CoV-2 spike S1 antibodies. As the participants were recruited from hospital workers they tended to be younger than the patients in the SARS-CoV-2 infection cohort.
Ethics oversight	The study was approved by the Cantonal Ethical Committee of Zurich (BASEC #2016-01440) and all participants signed a written informed consent before inclusion into the study.

Note that full information on the approval of the study protocol must also be provided in the manuscript.

Field-specific reporting

Please select the one below that is the best fit for your research. If you are not sure, read the appropriate sections before making your selection.

☒ Life sciences ☐ Behavioural & social sciences ☐ Ecological, evolutionary & environmental sciences

For a reference copy of the document with all sections, see nature.com/documents/nr-reporting-summary-flat.pdf

Life sciences study design

All studies must disclose on these points even when the disclosure is negative.

Sample size	The sample size for the SARS-CoV-2 Infection Cohort (n=65) was determined based on pre-experiments. For the SARS-CoV-2 Tonsil and Vaccination Cohorts the sample size was determined by sample availability.
Data exclusions	No patients were excluded from the analysis. For phenotypic analysis only samples with at least 10 SARS-CoV-2 spike-specific cells were included and for the spike-specific MBC subset analysis only if at least 4 per subset were recorded. Due to low dataset quality, one scRNA-seq dataset from the SARS-CoV-2 Infection Cohort was excluded from all gene expression analyses between groups of cells.
Replication	Samples were analysed once for the flow cytometry analysis due to sample availability. However in several batches, with longitudinal samples always in the analysed in the same batches. For the scRNA-seq experiments the tonsil and vaccination cohort datasets were acquired in 1

	batch respectively, for the SARS-CoV-2 infection cohort the samples were acquired in several batches including repetitions and subsequently integrated. The results of the repetitions were comparable.
Randomization	Not applicable as this is an observational study.
Blinding	As the patients were included based on the SARS-CoV-2 infection history, blinding could not performed.

Reporting for specific materials, systems and methods

We require information from authors about some types of materials, experimental systems and methods used in many studies. Here, indicate whether each material, system or method listed is relevant to your study. If you are not sure if a list item applies to your research, read the appropriate section before selecting a response.

Materials & experimental systems

n/a	Involved in the study	
☐	☒	Antibodies
☒	☐	Eukaryotic cell lines
☒	☐	Palaeontology and archaeology
☒	☐	Animals and other organisms
☒	☐	Clinical data
☒	☐	Dual use research of concern

Methods

n/a	Involved in the study	
☒	☐	ChIP-seq
☐	☒	Flow cytometry
☒	☐	MRI-based neuroimaging

Antibodies

Antibodies used

All Flow Cytometry and TotalSeq antibodies (antigen, fluorophore, provider, dilutions and cat no) used in the study are indicated in the supplementary tables 5-7 as part of the full panels.

Validation

Dilutions were determined for the antibodies in the lab by serial titrations, for TotalSeq antibodies the concentration were determined by titration of the corresponding flow cytometry antibody as suggested by the manufacturer. The antibodies were validated by the respective manufacturer (see below).

Biolegend:
- Flow Cytometry: The producers tests specificity on 1-3 target cell types with either single- or multi-color analysis (including positive and negative cell types). All the antibodies used had a verified human reactivity. Stainings of human PBMCs are shown (for anti-FcRL4 Cat No 340205 staining to a transfected cell line is shown and for anti-BCL-6 Cat No. 358511 to a Ramos lymphoma cell line) as flow plots or histograms on the website. Further each new lot is tested to perform with similar intensity to the in-date reference lot. Brightness (MFI) is evaluated from both positive and negative populations. Each lot product is validated by QC testing with a series of titration dilutions.
- Total-Seq: Biolegend test bulk lots by PCR and sequencing to confirm the oligonucleotide barcodes. They are also tested by flow cytometry to ensure the antibodies recognize the proper cell populations. Bottled lots are tested by PCR and sequencing to confirm the oligonucleotide barcodes.

BD Bioscience (Flow Cytometry):
Tested for flow cytometry application and verified human reactivity. Stainings of human PBMCs are shown as flow plots or histograms on the website for BD Horizon antibodies (anti- CD11c Cat No 612967, anti-IgD Cat No 566187, anti-Blimp1 Cat No 565274, anti-Ki67 Cat No 564071).

Invitrogen (Flow Cytometry):
Antibodies (antigen, fluorophore, dilution, Cat No): IRF8 V450 1/100 48-9852-82, CD45RB 1 APC 1/100 MA1-19461
Tested for flow cytometry application and verified human reactivity.

Miltenyi Biotec (Flow Cytometry):
Antibodies (antigen, fluorophore, dilution, Cat No): IgA APC 1/400 130-113-472 and PerCP-Vio770 1/400 130-114-004
Species reactivity human, QC tested and extended validation for specificity with epitope competition assay and sensitivity by performance comparison.

Cytognos (Flow Cytometry):
Antibodies (antigen, fluorophore, dilution, Cat No): IgG1 PE Cytognos 1/200 CYT-IGG1PE and IgG3 FITC Cytognos 1/200 CYT-IGG3F
Both designed for flow cytometry use as a direct immunofluorescence reagent in the identification and enumeration of IgG1 resp. IgG3 expressing cells. The products have been manufactured in accordance with standards of production and quality system of the ISO 13485:2016 and ISO 9001:2015 standards.

March 2021

Flow Cytometry

Plots

Confirm that:

☒ The axis labels state the marker and fluorochrome used (e.g. CD4-FITC).

☒ The axis scales are clearly visible. Include numbers along axes only for bottom left plot of group (a 'group' is an analysis of identical markers).

☒ All plots are contour plots with outliers or pseudocolor plots.

☒ A numerical value for number of cells or percentage (with statistics) is provided.

Methodology

Sample preparation	Blood was collected from patients and subsequently PBMCs were isolated using a Ficoll density gradient centrifugation, before being washed, counted and frozen in fetal bovine serum (FBS) with 10% dimethyl sulfoxide (DMSO) and stored in liquid nitrogen until use. Tonsils were mechanically cut into smaller pieces, grinded through a 70 micrometer cell strainer, washed in phosphate buffered saline, before a density gradient centrifugation was performed. Subsequently, the mononuclear cells were washed, counted, frozen in FBS with 10% DMSO and stored in liquid nitrogen until use. For subsequent analysis the frozen cells were thawed in pre-warmed R10 medium and subsequently processed for flow cytometry as described in the methods section.
Instrument	Samples were acquired on a Cytek Aurora and sorting was performed on an BD Aria III 4L.
Software	Flow cytometry data was generated using Cytek SpectroFlo (Version 3.0.3) and for sorting using BD FACSDiva (Version 8.0.1). The flow cytometry data were analysed using FlowJo (version 10.8.0).
Cell population abundance	As the cell numbers were low after sorting, all of the cells were loaded and processed for scRNA-sequencing using the 10x system. The cell identities were subsequently confirmed by single cell sequencing including feature barcoding.
Gating strategy	The full gating and sorting strategies are shown in the Extended Data Figures 2, 5 and 6.

☒ Tick this box to confirm that a figure exemplifying the gating strategy is provided in the Supplementary Information.
